# Balanced Flame Retardancy and Mechanical Enhancement of Epoxy Enabled by Low-Loading N-P-Si Modified ATH

**DOI:** 10.3390/polym18151890

**Published:** 2026-07-31

**Authors:** Ley Boon Sim, Jia Han, Yongming Zeng, Haoqi Wang, Yujia Qin, Weiwei Wang, Haiping Yang, Aygul Kadir

**Affiliations:** 1 School of Chemistry and Chemical Engineering, Changji University, Changji 831100, China; h1783698495@163.com (J.H.);; 2 School of Energy and Chemical Engineering, Xiamen University Malaysia, Jalan Sunsuria, Bandar Sunsuria, Sepang 43900, Malaysia; pce2311001@xmu.edu.my; 3 Department of Chemical and Biochemical Engineering, College of Chemistry and Chemical Engineering, Xiamen University, Xiamen 361005, China; 4 College of Chemistry and Environmental Engineering, Xinjiang Institute of Engineering, Urumqi 830091, China

**Keywords:** epoxy resin, ATH flame retardant, nitrogen-phosphorus-silicon hybridization, smoke suppression, synergistic effect

## Abstract

Numerous previous investigations have exploited single-component aluminum hydroxide, silica, or phosphorus-containing organic agents to improve the fire resistance of epoxy resin. Existing literature confirms that ATH relies on endothermic dehydration and inorganic barrier layers to suppress combustion, while phosphorus organics exert radical quenching effects in the gas phase. However, separate use of these fillers generally requires high loading to achieve satisfactory flame retardancy, which inevitably weakens the mechanical properties of the epoxy matrix; few studies integrate N, P, and Si elements into ATH via chemical grafting to realize synergistic flame retardancy at low filler dosage, and the dual heat-transfer regulation effect of formed SiO_2_-Al_2_O_3_ inorganic residues has rarely been systematically discussed in prior reports. This study presents an organic–inorganic hybrid flame retardant, SPDP-PTMS@ATH, synthesized by grafting N,P,Si-containing organic groups onto Al(OH)_3_. The modified ATH retained its layered structure, as confirmed by FTIR, XPS, SEM, and XRD. At only 5 wt.% loading in epoxy, the additive significantly enhanced flame retardancy and smoke suppression: LOI increased to 33.5% (34% higher than pure EP), UL-94 reached V-0 rating, and peak HRR, THR, COPR, TSR, CO2PR, and SPR are reduced by 30.2%, 30.8%, 33.1%, 26.9%, 25.86%, and 15%, respectively. Char analysis revealed a denser, more graphitized structure with fewer defects. Moreover, tensile strength and elongation at break improved by 22.0% and 47.5%, respectively. This work demonstrates that low-loading SPDP-PTMS@ATH simultaneously boosts fire safety, smoke suppression, and mechanical performance, offering a cost-effective and sustainable route to high-performance epoxy composites.

## 1. Introduction

Epoxy resins, valued for their strength, stability, designability, and adhesion, are widely applied in electronics, automotive, aerospace fields, etc. [1,2,3,4,5]. However, their carbon chains and epoxy groups confer a low limiting oxygen index (LOI), making them flammable and thus prone to releasing smoke, heat, and toxic gases. Improving flame retardancy is therefore critical for fire safety [6]: ideally, heat release rate (HRR) and total heat release (THR) should be reduced by ≥30%, LOI should reach ≥26% to avoid sustained combustion in air, and a UL-94 rating of V-1 or above indicates reliable self-extinguishing behavior with limited dripping. In addition to inherent flammability, fully crosslinked epoxy thermosets also suffer from well-known recycling limitations, since their irreversible covalent networks cannot be remelted and recycled through simple physical methods, while available chemical recycling approaches are costly and may damage polymer performance. Extending the service life of epoxy products by improving their fire safety with low-loading high-performance flame retardants can partly relieve such recycling pressure, which serves as one practical background motivation of this work.

Flame retardants are classified into organic types—halogen-, phosphorus-, nitrogen-, boron-, and silicon-based—and inorganic types such as metal hydroxides, oxides, and borates. Organic systems are generally more effective due to their multi-phase mechanisms: halogenated agents suppress combustion via radical quenching but are largely abandoned for toxic byproducts [7]; phosphorus-based promote char and release PO· radicals to lower HRR/THR but often suffer poor hydrolytic stability; nitrogen-based release inert gases that dilute oxygen and volatiles, raising LOI but show limited effect on other metrics [8,9,10]; silicon-based form a dense Si-O layer reinforcing char, improving thermal stability and UL-94 rating, though costly [11]; boron-based melt into glassy borates that integrate with char to form a compact barrier, enhancing LOI and THR with low toxicity and smoke suppression, but they are susceptible to thermal decomposition, and suffer from poor compatibility with the polymer substrate and adjacent additives [12].

Inorganic flame retardants are low-cost, environmentally benign, and low-toxicity, but show poor compatibility with other additives, and their effect is often limited to a single mechanism, limiting their overall fire protection efficiency compared with multifunctional flame-retardant systems. Even more so, single fillers often rely on high loadings, which impair dispersion and mechanical integrity, leaving them with inadequate overall performance [13].

Due to the limitations of single-component systems, current research increasingly emphasizes hybrid flame retardants, which combine organic and/or inorganic components to leverage complementary mechanisms for synergistic enhancement [14,15].

In organic-organic hybrids, P-N systems act synergistically where phosphorus participates in radical scavenging and promotes char formation, while nitrogen releases inert gases and stabilizes the char barrier. Although effective, many P-N systems demand high loadings (e.g., >15 wt.%) to achieve UL-94 V-0 and LOI ≈ 30%, and their impact on THR reduction is often modest [16,17].

P-Si systems act via the phosphorus component, which eliminates free radicals and accelerates carbonization, while silicon forms Si-O layers that densify char and may improve interfacial compatibility. Typical results show moderate HRR/THR reductions (~20–30%) and LOI values around 28–30%, though these are only marginally above the minimum requirement [18,19].

B-Si hybrids enhance protective stability by forming glassy borate coatings and Si-O networks that densify char. While such systems can achieve UL-94 V-0 and LOI ≈ 30% with some smoke suppression, reported HRR/THR reductions remain limited (~12–28%), and the required loadings are not always specified [20,21].

Overall, binary hybrids demonstrate significant synergistic benefits, yet many rely on relatively high dosages and deliver unbalanced performance across different metrics. Ternary systems, by integrating three elements, aim to extend these synergies: for instance, HBNPSi and PmDOH have been shown to achieve UL-94 V-0 and LOI ≥ 30%, but still with constrained THR reductions (<30%) at loadings around 10% or higher [22,23].

In summary, organic-organic hybrids provide pathways to improved efficiency and reduced toxicity, but they frequently require high loadings or lack clearly defined optimal dosages. While they commonly achieve UL-94 V-0 and LOI values above 30%, limitations in THR reduction and practical applicability remain. In contrast, inorganic-inorganic systems have been far less explored and generally suffer from mechanical deterioration and the need for very high loadings, underscoring the need for alternative strategies [24].

Organic–inorganic hybrids combine the high efficiency of organics with the low toxicity, heat resistance, and smoke suppression of inorganics. Yan [25] reported APEA/ATH with HRR and THR reductions of 38% and 29%, though loading was unspecified. Zhang [26] achieved UL-94 V-0 and LOI 40.3% in PBS using as high as 22% IFR and 3% K-HAPCP. Li [27] found 13.3% MPKG in PU reduced HRR and THR by 63.6% and 38.8% but gave LOI only 21.5% (<26%). Yu [28] obtained UL-94 V-0, LOI 34.8%, and HRR/THR reductions of 79%/65% in EP with 15% CEUP and 3% OMMT. These findings highlight the progress of organic–inorganic hybrids, yet they still show the persistent trade-off between efficiency and loading. Therefore, our goal is to develop a low-loading flame retardant with overall balanced performance and enhanced safety by synergistically improving LOI, HRR, and THR to meet comprehensive fire-resistance demands.

In this context, we noted that Liu [29] et al. synthesized an N-P-Si flame retardant (SPDP-PTMS), which raised cotton LOI to 29.5% with 22.7% and 32.3% reductions in HRR and THR at 500 g/L. Through sol–gel covalent bonding, it formed stable coatings with minimal structural damage, maintained tensile strength even at high concentration, and showed good durability (LOI 26.2% after 20 washes), overcoming the poor hydrolytic stability of phosphorus agents. Its multi-phase mechanism and mechanical compatibility suggest strong potential for epoxy systems, offering a new route for efficient flame-retardant modification.

However, N-P-Si systems still face poor polymer compatibility and limited stability at high temperature. To address these shortcomings, inorganic substrates can be introduced to provide complementary functions. ATH is particularly advantageous for hybrids as its surface hydroxyls allow grafting and its decomposition provides endothermic cooling, water vapor release, Al_2_O_3_ barrier formation, and inert filling.

However, conventional inorganic flame retardants such as pristine ATH usually suffer from poor dispersion, weak interfacial compatibility, and low flame-retardant efficiency at low loadings, which severely limit their practical application in high-performance epoxy composites. Fortunately, filler surface functionalization has been proven to be an effective strategy to solve these drawbacks. The introduction of organic functional groups containing phosphorus, nitrogen, and silicon can significantly improve the interfacial compatibility between inorganic fillers and epoxy matrix, avoid particle agglomeration, and construct multi-element synergistic flame-retardant systems. Therefore, rational functional modification of ATH provides a feasible and efficient way to develop high-performance epoxy composites with enhanced fire safety and mechanical properties.

Based on these insights, in this work, 4,4′-sulfonyldiphenol dicarbonate (SPDPC) was synthesized from POCl_3_ and pentaerythritol, further reacted with 3-(aminopropyl)trimethoxysilane (APTMS) to yield SPDP-PTMS, which was grafted onto ATH to prepare a novel SPDP-PTMS@ATH flame retardant and incorporated into epoxy (EP/SPDP-PTMS@ATH composites). The structure was thoroughly characterized by Fourier transform infrared (FTIR), X-ray diffraction (XRD), X-ray photoelectron spectroscopy (XPS), and scanning electron microscopy (SEM); thermal analysis by thermogravimetric analysis (TGA); flame retardancy by limiting oxygen index (LOI), vertical burning tests (UL-94), cone calorimetry (CCT); char inspection by SEM, Raman spectra (Raman), FTIR; resultant gas evaluation by thermogravimetry-FTIR (TGIR); and mechanical performance by tensile testing.

It should be noted that the synthesis of SPDPC employs POCl_3_, which introduces certain environmental drawbacks from a green chemistry perspective [30]. The present work focuses primarily on flame retardancy and related mechanisms, and therefore this aspect is not elaborated further.

## 2. Materials and Methods

### 2.1. Experimental Materials

Aluminum hydroxide (ATH, purity ≥ 99.0%, average particle size: 2–5 μm, moisture content ≤ 0.3%) was purchased from Sinopharm Chemical Reagent Co., Ltd. (Shanghai, China). Pentaerythritol was obtained from Chengdu Cologne Chemical Reagent Factory (Chengdu, China). Phosphorus oxychloride (POCl_3_) was supplied by Adamas Reagent Co., Ltd. (Shanghai, China). Absolute ethanol was obtained from Tianjin Xinbote Chemical Co., Ltd. (Tianjin, China). APTMS, triethylamine, and acetonitrile were purchased from Aladdin Biochemical Technology Co., Ltd. (Shanghai, China). Toluene was obtained from the Tianjin Chemical Reagent Research Institute (Tianjin, China). Epoxy resin E-44 (epoxy value of 0.41–0.47 mol/100 g and a relative molecular weight of approximately 450) was supplied by Hefei Jiangfeng Chemical Co., Ltd. (Hefei, China). 4,4′-Diaminodiphenylmethane (DDM) was purchased from Sinopharm Chemical Reagent Co., Ltd. (Shanghai, China). Diethyl ether was obtained from Tianjin Zhiyuan Chemical Reagent Co., Ltd. (Tianjin, China). Acetone was purchased from Shanghai Macklin Biochemical Co., Ltd. (Shanghai, China). Unless otherwise stated, all reagents and solvents were of analytical grade and used as received.

### 2.2. Synthesis of SPDPC and SPDP-PTMS

**Figure 1:**
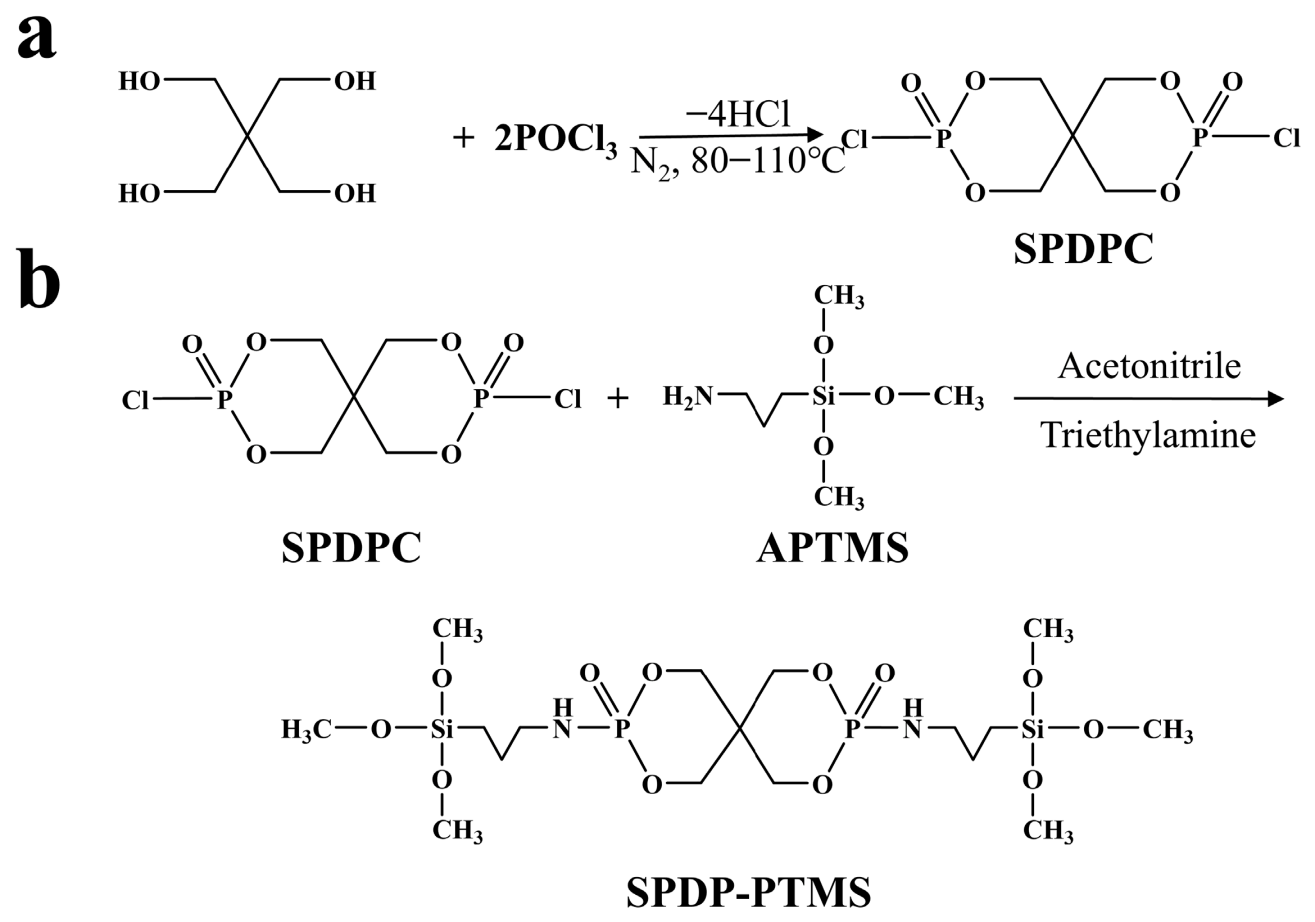
(**a**) Synthesis route of SPDPC, (**b**) Synthesis route of SPDP-PTMS.

Phosphorus oxychloride (POCl_3_, 0.55 mol, 84.33 g, M = 153.33 g/mol) and pentaerythritol (0.10 mol, 13.62 g, M = 136.15 g/mol) were charged into a three-necked flask under a nitrogen atmosphere. The mixture was heated to 80 °C and maintained until the reaction proceeded smoothly, after which the temperature was raised to 110 °C. The reaction was continued until no further evolution of HCl gas was observed (≈8 h) (Measured by pH test paper). Upon completion, the mixture was cooled to room temperature, washed with diethyl ether, and the solvent was removed by rotary evaporation. The crude product was further dried under vacuum at 60 °C for 24 h to afford a white powder (25.5 g, yield ≈ 86%). The reaction scheme is shown in Figure 1a [29,30,31,32].

SPDPC (2.97 g, 0.01 mol) and triethylamine (3 mL) were added to a three-necked flask containing acetonitrile (50 mL) as solvent. 3-Aminopropyltrimethoxysilane (APTMS, 11.67 g, 0.02 mol) was then introduced, and the reaction was carried out at room temperature under a nitrogen atmosphere. After completion, the mixture was filtered to remove triethylamine hydrochloride. The filtrate was concentrated by rotary evaporation, and the residue was subjected to vacuum distillation at 70 °C under a pressure of 0.08 MPa to yield a pale yellow oily liquid (13.27 g, yield ≈ 86%). The reaction scheme is shown in Figure 1b [29].

### 2.3. Preparation of SPDP-PTMS@ATH

**Figure 2:**
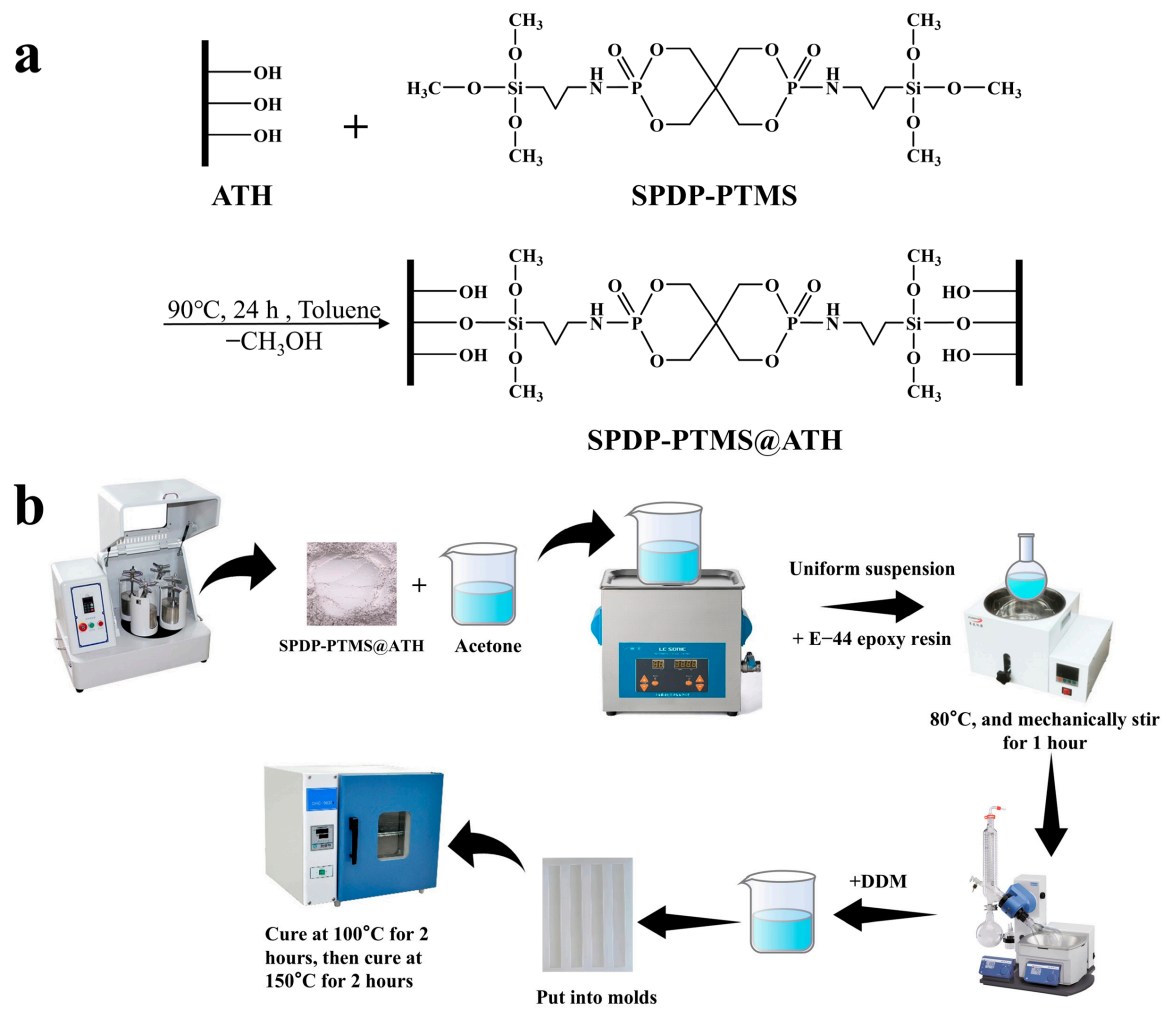
(**a**) Synthesis route of SPDP-PTMS@ATH, (**b**) Synthesis route of EP/SPDP-PTMS@ATH.

ATH (0.57 g) was dispersed in toluene and ultrasonicated for 0.5 h to obtain a uniform suspension. The suspension was then transferred into a three-necked flask and mixed with SPDP-PTMS (5.23 g). The reaction was carried out under a nitrogen atmosphere at 90 °C with reflux stirring for 24 h. The resulting solid was collected by filtration, washed three times with absolute ethanol, and dried under vacuum at 60 °C to yield the final product, denoted as SPDP-PTMS@ATH (yield ≈ 76%). The synthesis scheme is illustrated in Figure 2a.

### 2.4. Preparation of EP/SPDP-PTMS@ATH Composites

Epoxy composites containing different loadings of SPDP-PTMS@ATH were prepared by incorporating the modified filler into epoxy resin (EP). Prior to use, SPDP-PTMS@ATH was ground in a ball mill and passed through a 220-mesh sieve.

Using EP/5 wt.% SPDP-PTMS@ATH as an example: SPDP-PTMS@ATH (2.0 g) was dispersed in acetone (20 mL) by mechanical stirring, followed by ultrasonic treatment for 1 h to obtain a uniform suspension. This suspension was poured into molten E-44 epoxy resin (31.2 g) maintained at 80 °C in an oil bath, and mechanically stirred for 1 h. Since acetone readily volatilizes at this temperature, most solvent escaped during stirring, and the residual acetone was further removed in a separate evaporation process afterward. The mixture was then transferred to a single-neck flask and subjected to rotary evaporation for 0.5 h to completely remove the acetone. Subsequently, the curing agent 4,4′-diaminodiphenylmethane (DDM, 6.8 g) was added under continuous stirring until fully dissolved. The homogeneous mixture was cast into molds and cured at 100 °C for 2 h, followed by post-curing at 150 °C for 2 h. The cured composites were then demolded and stored for subsequent testing [33,34]. It should be noted that, for clarity and formatting purposes, the loading amount originally expressed in wt.% is simplified as % throughout this article. The synthesis scheme is illustrated in Figure 2b.

### 2.5. Characterization

Fourier transform infrared (FTIR) spectra were recorded on a Nicolet 6700 FTIR spectrometer (Thermo Fisher Scientific, Madison, WI, USA)over the wavenumber range of 600–4000 cm^−1^. X-ray diffraction (XRD) measurements were performed on a DX-2700 diffractometer (Dandong Haoyuan Instrument Co., Ltd., Dandong, China) with Cu Kα radiation (λ = 1.5418 Å) at 36 kV and 20 mA, covering a 2θ scanning range of 10–80° at a step size of 0.01°. X-ray photoelectron spectroscopy (XPS) was conducted on a Thermo ESCALAB 250Xi spectrometer (Thermo Fisher Scientific, Waltham, MA, USA) using a monochromatic Al Kα source (hν = 1486.6 eV). Scanning electron microscopy (SEM) images were acquired with a Hitachi SU3900 microscope (Hitachi High-Technologies Corporation, Tokyo, Japan) at an accelerating voltage of 10 kV, with all samples sputter-coated with a thin gold layer prior to observation. Thermogravimetric analysis (TGA) was carried out on a TG/DTG6300 thermal analyzer (SII Nanotechnology Inc., Chiba, Japan) under a nitrogen atmosphere (40 mL/min) from 30 °C to 800 °C at a heating rate of 20 °C/min. The limiting oxygen index (LOI) was measured using a 5810A automatic oxygen index tester (Vouch Testing Technology Co., Ltd., Suzhou, China) following GB/T 2406, with specimen dimensions of 100 × 7 × 3 mm^3^. Vertical burning tests (UL-94) were performed on a UL-94 horizontal/vertical burning tester (Vouch Testing Technology Co., Ltd., Suzhou, China) according to the UL-94 standard, using samples of 130 × 13 × 3 mm^3^. Cone calorimetry (CCT) was conducted on an FTT iCone mini cone calorimeter (Fire Testing Technology Ltd., East Grinstead, UK) in compliance with ISO 5660; samples (100 × 100 × 3 mm^3^) were wrapped in aluminum foil and exposed horizontally to an external heat flux of 50 kW/m^2^. Raman spectra were collected on an Xplora confocal Raman microscope (Horiba Scientific, Kyoto, Japan) over the range of 500–2000 cm^−1^, with burned char residues mounted on glass slides. FTIR spectra of char residues were recorded on an IRAffinity-1 spectrometer (Shimadzu, Kyoto, Japan) from 600 to 4000 cm^−1^. Thermogravimetry-FTIR (TGIR) analysis was performed using a Netzsch STA-2500 thermal analyzer (Netzsch, Selb, Germany) coupled with a Thermo Fisher IS-50 FTIR spectrometer (Thermo Fisher Scientific, Madison, WI, USA) under nitrogen flow (45 mL/min), heated from 30 °C to 800 °C at 20 °C/min. Tensile tests were carried out on an AL-7000MN high-speed servo-controlled universal testing machine (Gotech Testing Machines, Taichung, Taiwan, China) following ASTM D3039-08, with at least five parallel specimens tested per formulation to obtain average values.

## 3. Results and Discussion

### 3.1. Structural Characterization

**Figure 3:**
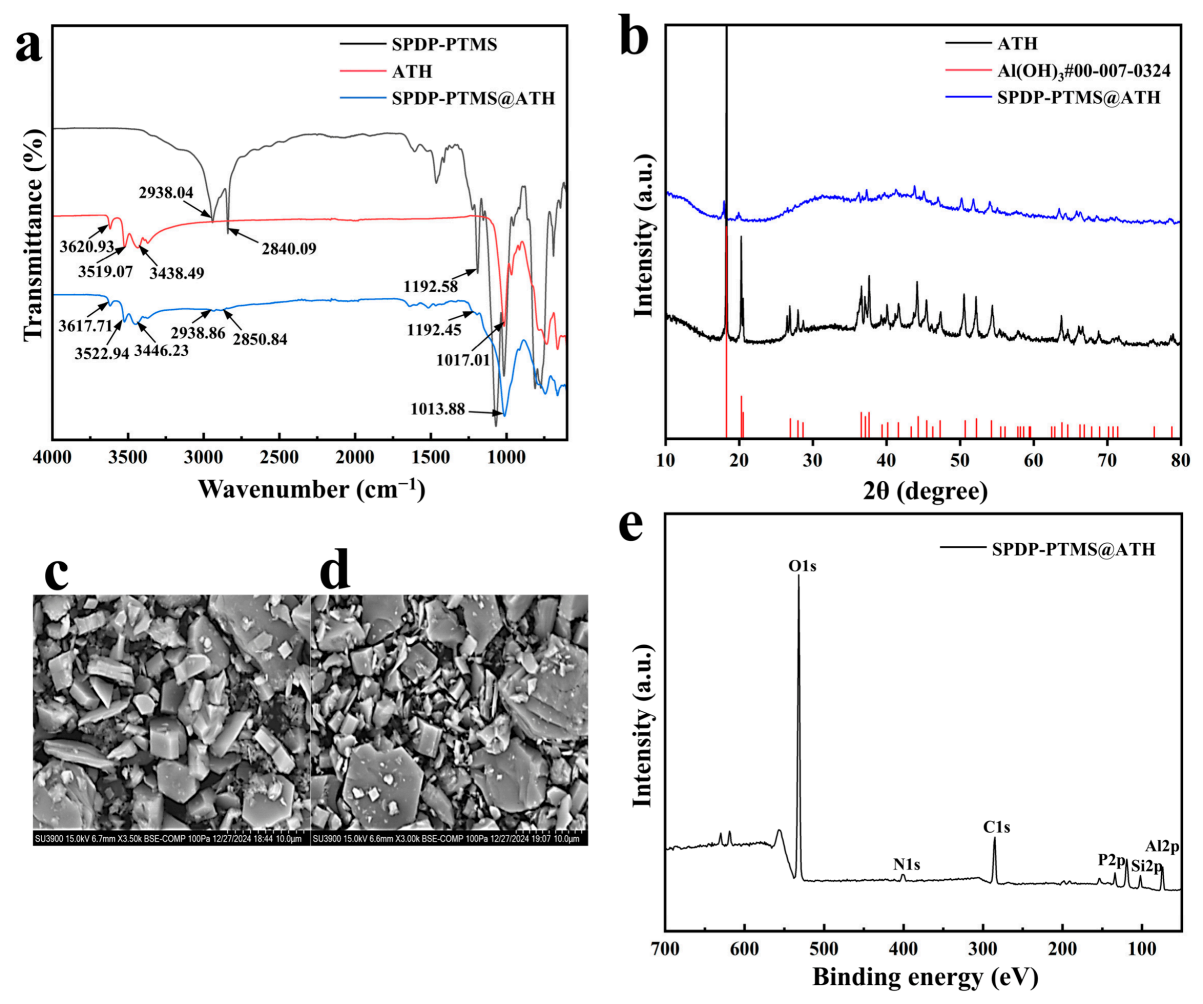
(**a**) Infrared spectra of SPDP-PTMS, ATH, and SPDP-PTMS@ATH, (**b**) XRD spectra of standard cards for ATH, SPDP-PTMS@ATH, and ATH, SEM images of (**c**) ATH and (**d**) SPDP-PTMS@ATH, and (**e**) XPS total spectra of SPDP-PTMS@ATH.

FTIR analysis was conducted to identify the characteristic absorption bands of functional groups in SPDP-PTMS, ATH, and SPDP-PTMS@ATH, as shown in Figure 3a. For SPDP-PTMS, the absorption bands at 2938.04 and 2840.09 cm^−1^ correspond to the C-H stretching vibrations of -CH_3_ and alkyl -CH_2_- segments in its molecular chain, respectively. The band at 1192.58 cm^−1^ is assigned to P-N stretching, and the peak at 1017.01 cm^−1^ to Si-O bending vibrations [29]. In the spectrum of ATH, the hydroxyl stretching vibrations are observed at 3620.93, 3519.07, and 3438.49 cm^−1^ [35]. After modification, SPDP-PTMS@ATH shows weakened hydroxyl absorption peaks at 3617.71, 3522.94, and 3446.23 cm^−1^. Meanwhile, the C-H stretching peaks of -CH_3_ and -CH_2_- at 2938.86 and 2850.84 cm^−1^ are also markedly reduced in intensity rather than fully disappearing. This phenomenon is attributed to the fact that SPDP-PTMS molecules are immobilized on the surface of ATH after grafting. The restricted molecular motion and reduced relative content of organic moieties suppress the C-H vibrational signals [36,37,38]. Additionally, the P-N stretching band at 1192.45 cm^−1^ and a Si-O bending peak at 1013.88 cm^−1^. These changes confirm the successful grafting of SPDP-PTMS onto ATH.

The XRD patterns of ATH, SPDP-PTMS@ATH, and the standard ATH card (Al(OH)_3_#00-007-0324) are presented in Figure 3b. The red line represents the ATH XRD standard card. ATH shows characteristic sharp diffraction peaks corresponding to the gibbsite polymorph of ATH [39]. After modification, the diffraction intensity decreases due to coverage by the amorphous SPDP-PTMS; the calculated interplanar distance of gibbsite shows no obvious change before and after treatment, demonstrating that SPDP-PTMS is mainly distributed on the surface of ATH rather than intercalated into its interlayer space. The characteristic reflections of ATH remain, indicating that the layered structure of ATH is preserved.

As shown in the SEM images of ATH and SPDP-PTMS@ATH (Figure 3c,d), the layered morphology of ATH remains unchanged after modification, indicating that the incorporation of SPDP-PTMS does not disrupt its crystalline structure. This observation is consistent with the XRD results, as gibbsite is inherently a layered hydroxide. It further demonstrates that the modification does not alter the physical characteristics of ATH, which retains its layered structure that intrinsically renders its good dispersibility within polymers, because its weak interlayer hydrogen bonding allows the layers to cleave or delaminate more readily under shear, improving compatibility and distribution compared with non-layered fillers.

XPS analysis (Figure 3e) was conducted to investigate the elemental composition of SPDP-PTMS@ATH. The main detected elements were C, O, N, P, Si, and Al, consistent with the designed hybrid structure. Quantitative analysis showed atomic percentages of 23.95% C, 49.36% O, 3.43% N, 2.89% P, 4.28% Si, and 16.09% Al, in reasonable agreement with the theoretical composition of SPDP-PTMS@ATH.

### 3.2. Thermogravimetric Analysis

**Figure 4:**
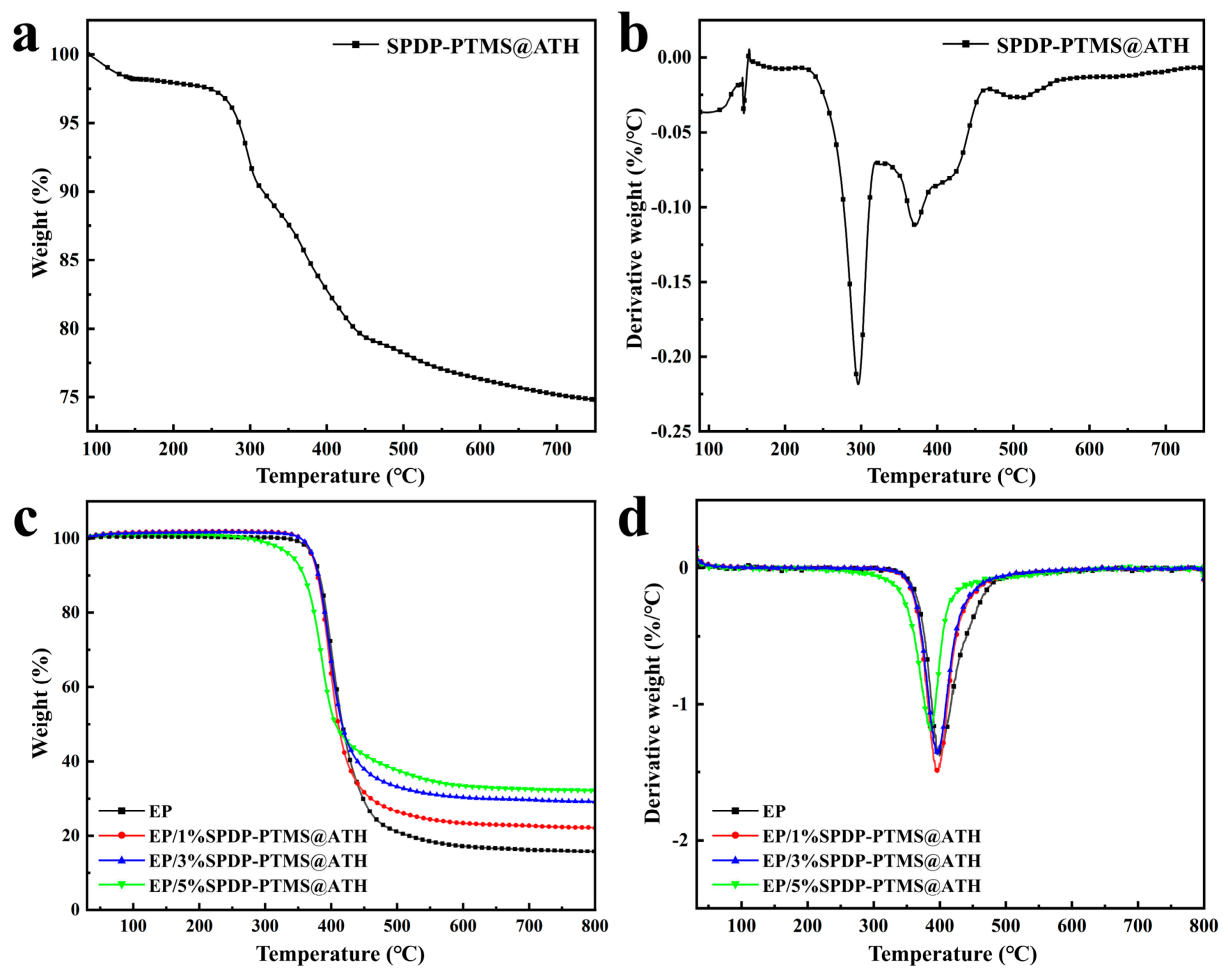
(**a**) TG and (**b**) DTG of SPDP-PTMS@ATH, and (**c**) TG and (**d**) DTG of EP with various SPDP-PTMS@ATH proportions.

Thermal analysis can reveal decomposition-related changes in SPDP-PTMS@ATH during combustion. Thermogravimetric analysis (TG) measures mass changes under heating, while derivative thermogravimetry (DTG) shows the rate of mass loss with temperature. SPDP-PTMS@ATH was subjected to thermal testing, as shown in Figure 4a,b. In addition, pure EP and EP composites containing different loadings of SPDP-PTMS@ATH were also analyzed, as shown in Figure 4c,d. The detailed results are summarized in Table 1.

The TG curve shows a slow decline and the DTG curve a slight rise between 100 and 140 °C, indicating minor mass loss at a decreasing rate. This behavior is attributed to the gradual evaporation of physically adsorbed water on the SPDP-PTMS@ATH surface.

Between 140–200 °C, the DTG curve shows a sharp drop at ~140 °C, a rebound near 145 °C, and approaches zero at 150 °C, forming a narrow spike that corresponds to slight weight loss in the TG curve. This feature arises from rapid desorption of free water from ATH surfaces and pores, a purely physical process without bond cleavage.

From 230–320 °C, the mass-loss rate rises sharply to a first peak, reflecting sudden weight loss in the TG curve. This stage corresponds to the endothermic O-H bond cleavage in ATH, releasing crystalline water [40], while phosphorus species form aluminum phosphate and amino groups decompose into nonflammable gases (NH_3_, H_2_O, CO_2_). During heating, the phosphorus-containing flame retardant decomposes and releases volatile phosphorus oxides and phosphorus-containing fragments. These volatile phosphorus products react with in-situ generated aluminum phosphate to facilitate char accumulation and form a compact protective layer, which effectively restrains the mass loss of the material [25]. Therefore, although after the peak the rate declines, it does not return to zero, showing that decomposition continues.

Between 330–550 °C, the TG curve declines steadily while the DTG curve shows a second peak. In 330–370 °C, a sharp drop reflects O-P-C bond cleavage in SPDP-PTMS, releasing phosphorus-containing volatiles (e.g., P oxides). Concurrently, aluminum phosphate derivatives react further to form orthophosphate aluminum, releasing H_2_O, NH_3_, and other small molecules [25,41]. From 370–465 °C, mass loss slows down, with a relatively stable interval (395–425 °C) attributed to orthophosphate aluminum reinforcing the char and forming a dense thermal barrier. At 465–550 °C, the mass-loss rate rises again, then decreases, mainly due to degradation of O=P groups. Above 550 °C, the TG curve stabilizes, and the DTG approaches zero, indicating that most components have decomposed, with only gradual graphitization or stabilization of residues at very low mass-loss rates [42].

Nonetheless, EP and its composites display similar thermal degradation with a single peak near 400 °C (Figure 4c,d), arising from C-C and C-O bond scission in the backbone and release of volatiles such as benzene and toluene [43]. Even with the addition of SPDP-PTMS@ATH, EP main-chain degradation dominates, leaving the overall decomposition pathway unchanged, which indicates that SPDP-PTMS@ATH does not alter the intrinsic thermal decomposition pathway of EP.

Figure 4c and Table 1 further show that increasing SPDP-PTMS@ATH content lowers the mass-loss rate and raises the char yield at 800 °C (CY_800_) from 15.8% for pristine EP to 32.0% at 5 wt.% SPDP-PTMS@ATH. This improvement arises from ATH dehydration to Al_2_O_3_ and SPDP-PTMS-induced char promotion, which together form a compact barrier that limits heat and volatile release, confirming the role of SPDP-PTMS@ATH during EP combustion.

To quantitatively analyze the synergistic carbon formation effect between SPDP-PTMS@ATH and the epoxy resin matrix, the theoretical carbon residue values of composite materials with different additive concentrations were calculated using a mass-weighted summation approach based on the carbon residue rates at 800 °C for pure epoxy resin (EP) and pure SPDP-PTMS@ATH. The calculation formula was:

CY_theoretical_ = (1 − w) × CY_EP_ + w × CY_SPDP-PTMS@ATH_ (1)

where w represents the mass fraction of SPDP-PTMS@ATH, CY_EP_ = 15.8%, and CY_SPDP-PTMS@ATH_ = 74.8% are the high-temperature carbon residue rates of pure epoxy resin and pure composite filler, respectively.

Calculations revealed that the theoretical carbon residues for systems containing 1%, 3%, and 5% EP/SPDP-PTMS@ATH were 16.39 wt.%, 17.57 wt.%, and 18.75 wt.%, all significantly lower than the measured values of 22.0 wt.%, 29.1 wt.%, and 32.0 wt.% for the same formulations. These theoretical values reflect only the simple physical sum of the two components in the absence of interaction, whereas the measured carbon residues substantially exceed the theoretical predictions, demonstrating a pronounced synergistic carbon formation effect between SPDP-PTMS@ATH and the epoxy matrix.

Higher SPDP-PTMS@ATH loadings resulted in reduced DTG peak intensity, indicating lower maximum mass-loss rates (R_max_) (Figure 4d, Table 1). At 1–3 wt.%, the effect on onset stability is minor, though char forms earlier and decomposition ends sooner. At 5 wt.% loading, decomposition shifts further left in TG/DTG curves, reflecting earlier decomposition, which encourages protective char formation sooner, before the material can burn intensively. Along with the lowest peak intensity, it collectively best helps limit heat release, flame spread, and fire risk.

### 3.3. Flame-Retardant Performance

#### 3.3.1. LOI and UL-94 Tests

LOI and UL-94 tests were conducted to evaluate the flammability of epoxy composites and the self-extinguishing capability of EP, EP/1%SPDP-PTMS@ATH, EP/3%SPDP-PTMS@ATH, and EP/5%SPDP-PTMS@ATH, and the results are summarized in Table 2. Pristine EP is highly flammable, with an LOI of only 25% and no UL-94 vertical rating, accompanied by dripping during burning [44]. Upon addition of SPDP-PTMS@ATH, the LOI values increased to 27.3%, 29.8%, and 33.5% for 1, 3, and 5 wt.% loadings, respectively. Correspondingly, the UL-94 ratings improved to V-1, V-1, and V-0, with no dripping observed. These results clearly demonstrate that SPDP-PTMS@ATH effectively enhances both oxygen resistance and vertical burning performance of EP, achieving a V-0 rating at only 5 wt.% loading.

In practical applications, flame-retardant dosage is typically determined based on compliance with industry-standard benchmarks (e.g., UL-94, GB/T 2408), where achieving LOI ≥ 28% and a V-0 rating is considered eligible for electronic, electrical, and construction materials. The present results indicate that SPDP-PTMS@ATH can meet these thresholds at relatively low loadings, aligning with the “low-addition, high-performance” design strategy. This ensures that the developed system not only meets key safety requirements but also offers practical advantages for industrial application.

#### 3.3.2. Cone Calorimetry

**Figure 5:**
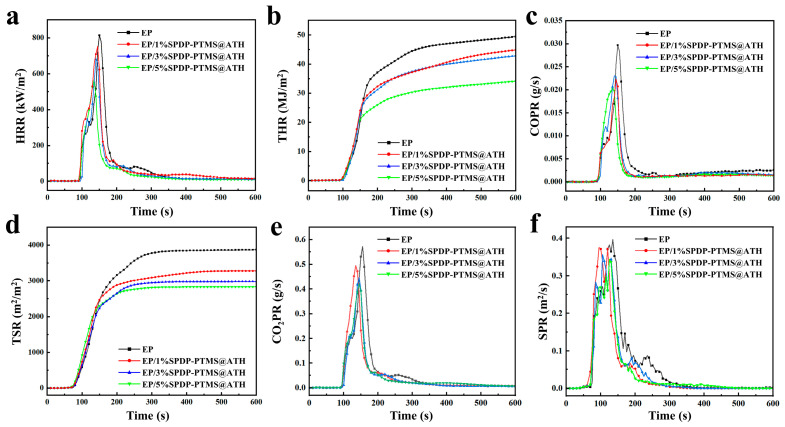
(**a**) HRR, (**b**) THR, (**c**) COPR, (**d**) TSR, (**e**) CO_2_PR, and (**f**) SPR of EP and EP/SPDP-PTMS@ATH.

To further evaluate the heat release and combustion behavior of EP after SPDP-PTMS@ATH incorporation, cone calorimeter (CCT) tests were conducted on pristine EP and the EP composites. Figure 5a–d and Table 3 further present the time to ignition (TTI), heat release rate (HRR), total heat release (THR), CO production rate (COPR), total smoke release (TSR), CO_2_ production rate (CO_2_PR), and smoke production rate (SPR) of pristine EP and EP composites.

It can be seen from TTI results that the epoxy composite filled with 3 wt.% SPDP-PTMS@ATH possesses superior ignition resistance, while the specimens with 1 wt.% and 5 wt.% filler loadings exhibit nearly identical time to ignition to neat epoxy, proving that this composite filler will not deteriorate the anti-ignition property of the epoxy matrix. Pristine EP exhibits severe flammability, with an HRR of 814 kW/m^2^, THR of 49.4 MJ/m^2^, COPR of 0.0296 g/s, and TSR of 3870 m^2^/m^2^, indicating intense heat and smoke release during burning. In contrast, incorporation of SPDP-PTMS@ATH significantly suppresses these parameters, with greater improvements at higher loadings. At 5 wt.% addition, HRR, THR, COPR, TSR, CO_2_PR and SPR are reduced by 30.2%, 30.8%, 33.1%, 26.9%, 25.86% and 15%, respectively, confirming the strong flame-retardant efficiency of this multi-element synergistic system.

The flame-retardant suppression originates from coordinated gas-phase and condensed-phase effects. ATH undergoes endothermic decomposition to release water vapor and consume ambient heat; meanwhile, the degradation of SPDP-PTMS yields inert CO_2_, N_2_, and NH_3_ to dilute oxygen and flammable pyrolysis volatiles. In the gas phase, phosphorus fragments generate phosphorus radicals to terminate combustion chain reactions, whereas they transform into phosphoric and polyphosphoric acidic oxides in the condensed phase to accelerate char generation. Silicon and aluminum constituents further construct inert SiO_2_ and Al_2_O_3_ layers within char residues, strengthening the char framework into a compact barrier that blocks heat and oxygen transport. Such multi-element synergies lower the composites’ HRR, THR, COPR, TSR, CO_2_PR, and SPR, and deliver gradually enhanced fire resistance as the dosage of SPDP-PTMS@ATH rises.

To further reveal the smoke toxicity and combustion oxidation state during combustion, the CO/CO_2_ ratio of each sample was calculated and analyzed to interpret the underlying flame-retardant mechanisms. The neat epoxy shows a CO/CO_2_ ratio of 0.0519, while the ratios of EP/1%, EP/3% and EP/5% SPDP-PTMS@ATH composites decline to 0.0455, 0.0513 and 0.0471, respectively. All modified composites deliver lower or comparable CO/CO_2_ values relative to pristine EP, which demonstrates more sufficient oxidation and reduced toxic CO release after adding SPDP-PTMS@ATH. The water vapor released by endothermic decomposition of ATH dilutes flammable volatiles and promotes full oxidation of pyrolytic fragments, inhibiting the generation of toxic CO via incomplete anoxic combustion. Meanwhile, the P-Si-N segments in SPDP-PTMS jointly suppress CO emission through condensed-phase char barrier and gas-phase radical quenching effects, which provides direct evidence for the dual-gas-condensed-phase synergistic flame-retardant mechanism of SPDP-PTMS@ATH from the perspective of smoke toxicity.

(2) $$FRI=\frac{{\left[\left(\frac{pHRR}{t_{i}}\right)\times THR\right]}_{Neat\;Polymer}}{{\left[\left(\frac{pHRR}{t_{i}}\right)\times THR\right]}_{Composite}}$$

In this formula, *t_i_* denotes time to ignition (TTI), and pHRR represents peak heat release rate. The numerator corresponds to neat epoxy resin, while the denominator stands for SPDP-PTMS@ATH filled composite samples [45].

To further quantify the overall fire resistance of each composite, the flame retardancy index (FRI) proposed by Vahabi et al. was calculated according to Equation (2), which integrates pHRR, TTI and THR to comprehensively rank the flame-retardant efficiency of polymer materials. The calculated FRI values of EP/1%, EP/3% and EP/5% SPDP-PTMS@ATH composites are 1.25, 1.45 and 2.04, respectively. All modified samples deliver FRI values greater than 1, which quantitatively confirms that the incorporation of SPDP-PTMS@ATH effectively improves the comprehensive flame retardancy of epoxy resin, and the fire safety performance is continuously enhanced with the increase of filler loading. This quantitative ranking result is consistent with the variation trend of HRR, THR, and smoke-related parameters above, further verifying the outstanding multi-element synergistic flame-retardant effect of SPDP-PTMS@ATH.

In addition, we will collect residual carbon obtained from the combustion of EP, EP/1%SPDP-PTMS@ATH, EP/3%SPDP-PTMS@ATH, and EP/5%SPDP-PTMS@ATH after conical calorimetry for testing. The following macroscopic morphology analyses of residual carbon, SEM characterization, Raman analysis, and infrared analysis of residual carbon are all generated by conical calorimetry tests.

#### 3.3.3. Residual Char Morphology Analysis

To further probe the flame-retardant mechanism of SPDP-PTMS@ATH in EP, the macroscopic morphology and SEM images of char residues after CCT were examined.

**Figure 6:**
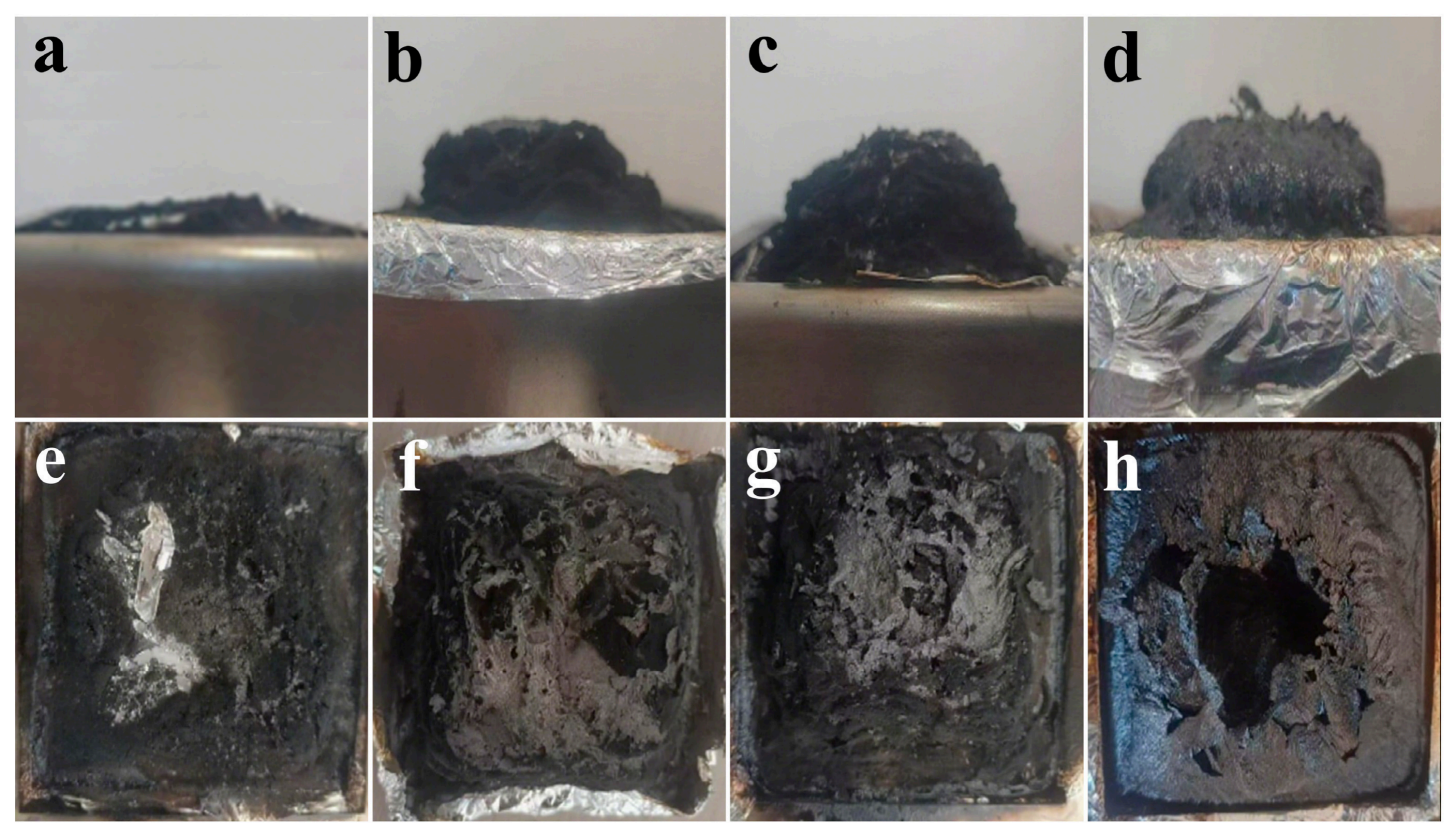
Carbon residue side views of (**a**) EP, (**b**) EP/1%SPDP-PTMS@ATH, (**c**) EP/3%SPDP-PTMS@ATH, and (**d**) EP/5%SPDP-PTMS@ATH and their (**e**,**f**,**g**,**h**) top view counterparts in the same sequence, respectively.

As shown in Figure 6a–d, pristine EP leaves little, fragile char, whereas EP with 1–5 wt.% SPDP-PTMS@ATH produces more expanded, denser residues, with EP/5% showing the most swelling and compact surface; this aligns with the TG observation that increasing the SPDP-PTMS@ATH content results in higher residual mass. We further observed the char expansion ratio of each sample: pure EP presents an extremely low expansion ratio, while EP composites filled with 1–5 wt.% SPDP-PTMS@ATH deliver remarkably improved expansion performance. The expansion ratio rises gradually with the loading of SPDP-PTMS@ATH, and EP/5%SPDP-PTMS@ATH achieves the maximum expansion ratio as well as the densest char surface. This improvement arises from the six-membered ring structures of the additive releasing nonflammable gases that dilute oxygen and drive char expansion, while P-O bonds generate phosphorus acids that catalyze matrix charring. Meanwhile, Si and Al form SiO_2_ and Al_2_O_3_ layers that further densify the char, creating an effective thermal and oxygen barrier.

**Figure 7:**
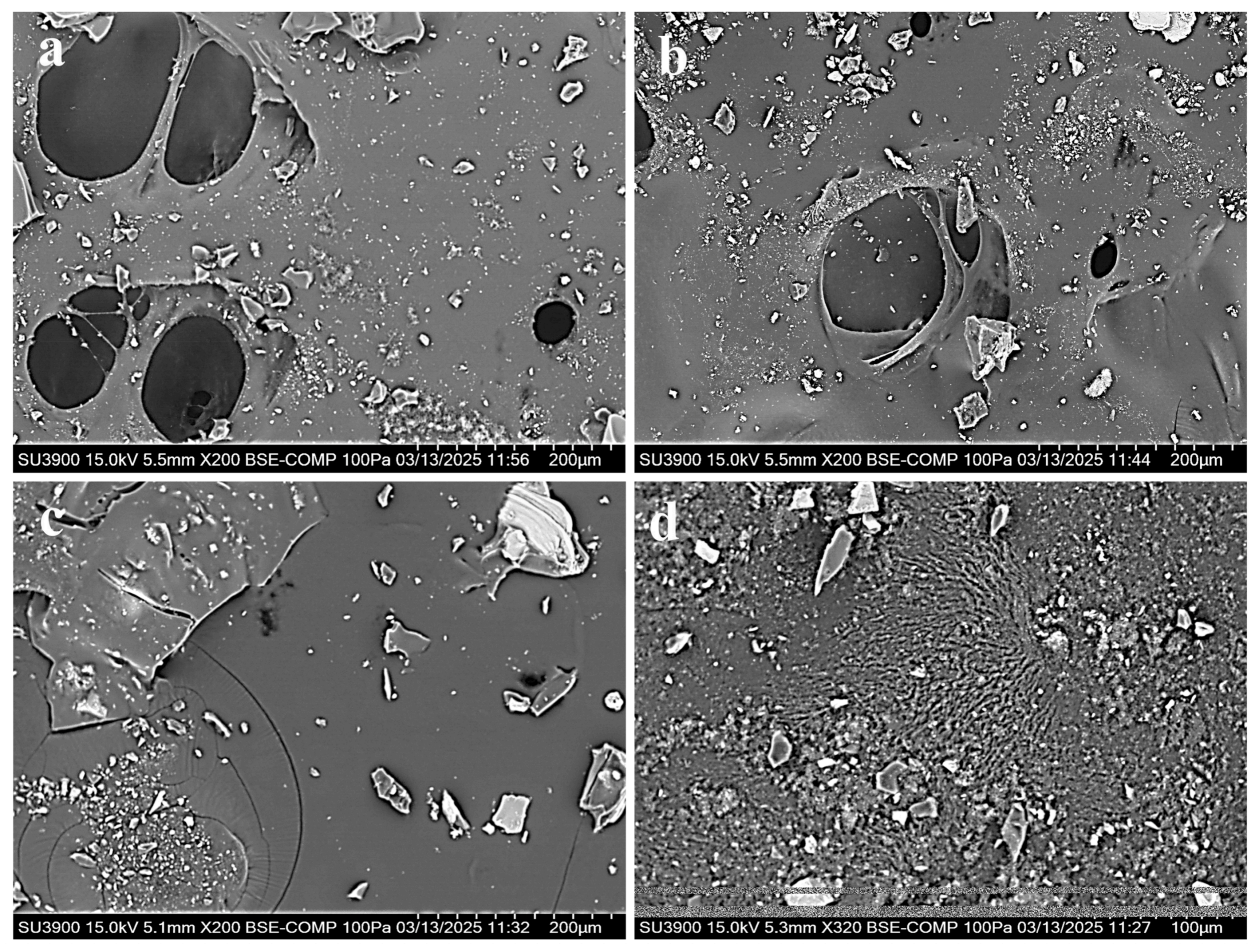
SEM images of intumescent char residues of (**a**) EP, (**b**) EP/1%SPDP-PTMS@ATH, (**c**) EP/3%SPDP-PTMS@ATH, and (**d**) EP/5%SPDP-PTMS@ATH after combustion.

SEM images of the char (Figure 7a–d) confirm these observations. The residue of pristine EP (Figure 7a) is fractured, porous, and riddled with voids, indicating that it cannot function as an effective barrier; combustible volatiles easily escape, sustaining vigorous burning. By contrast, the char from EP composites (Figure 7b–d) becomes progressively more continuous and compact as the SPDP-PTMS@ATH content increases. In particular, EP/5%SPDP-PTMS@ATH (Figure 7d) exhibits a smooth, dense surface with minimal cracks or voids. This dense char significantly strengthens the surface barrier, limiting heat transfer and volatile release, thereby suppressing flame propagation and accelerating self-extinguishment.

Overall, both macroscopic and microscopic analyses demonstrate that SPDP-PTMS@ATH confers strong condensed-phase flame retardancy by promoting the formation of a dense, stable, and excellently intumescent char layer, which interrupts combustion and increases residual char yield in EP composites.

#### 3.3.4. Residual Char Raman Analysis

**Figure 8:**
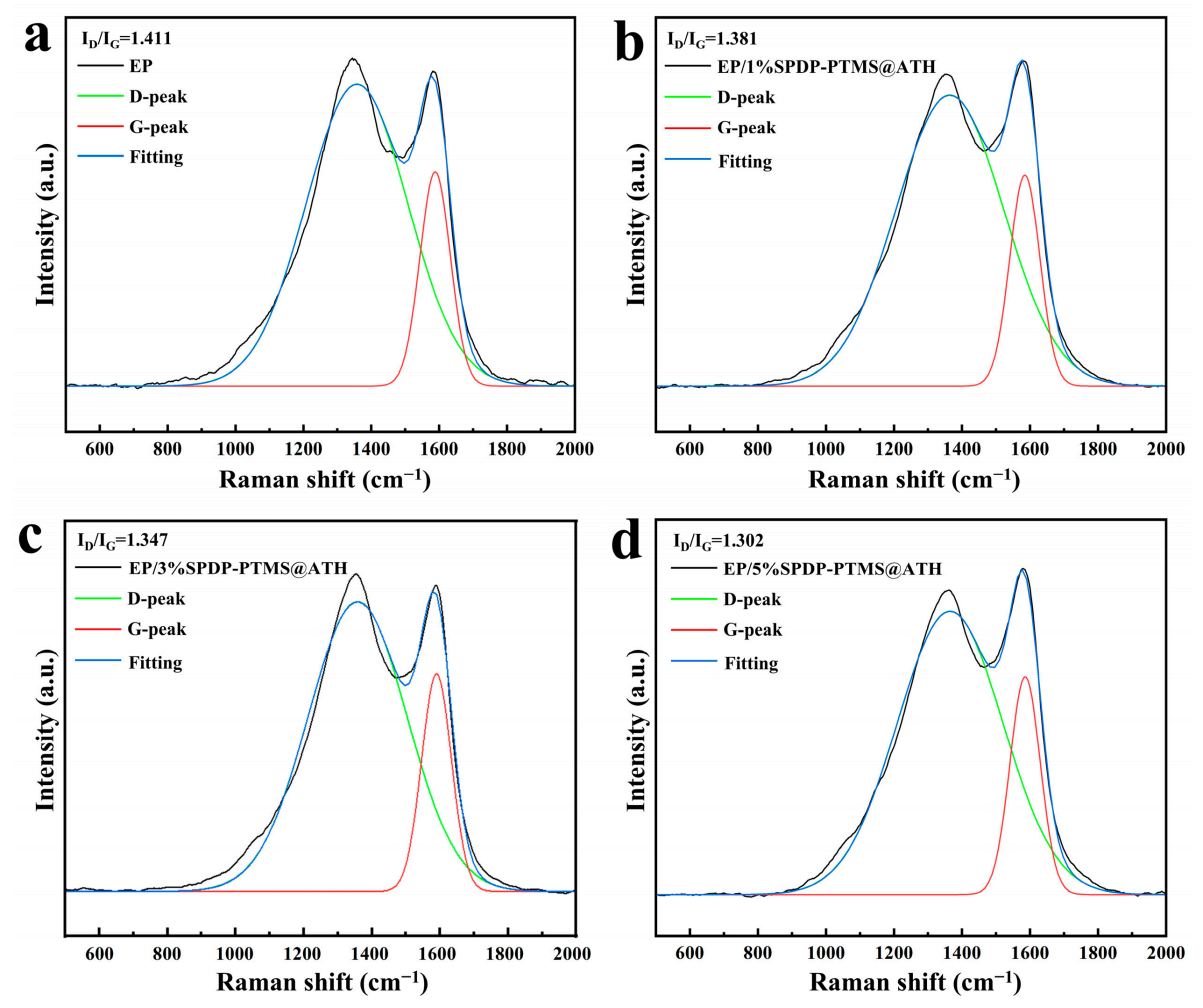
Raman spectra of intumescent char residues of (**a**) EP, (**b**) EP/1%SPDP-PTMS@ATH, (**c**) EP/3%SPDP-PTMS@ATH, and (**d**) EP/5%SPDP-PTMS@ATH after combustion.

Raman spectroscopy was performed to examine the graphitization degree of the residual char from pristine EP, EP/1%SPDP-PTMS@ATH, EP/3%SPDP-PTMS@ATH, and EP/5%SPDP-PTMS@ATH after combustion (Figure 8a–d). In Raman spectra, the D band (~1350 cm^−1^) corresponds to disordered carbon, while the G band (~1580 cm^−1^) represents graphitic carbon. The intensity ratio (I_D_/I_G_) is commonly used to assess graphitization: lower values indicate higher graphitic order, which enhances thermal stability and barrier performance [46,47,48,49].

The I_D_/I_G_ ratios for EP, EP/1%, EP/3%, and EP/5% SPDP-PTMS@ATH are 1.411, 1.381, 1.347, and 1.302, respectively, showing a progressive decrease with increasing additive content. This trend confirms that SPDP-PTMS@ATH promotes carbon atom rearrangement during decomposition, producing more ordered graphite-like structures. The resulting dense, thermally stable char layer improves thermal and oxygen insulation within the matrix, causing combustion to gradually cease. Nonetheless, this test also corroborates the earlier conclusions drawn from both macroscopic observation and SEM analysis of the char residue.

#### 3.3.5. Residual Char FTIR Analysis

**Figure 9:**
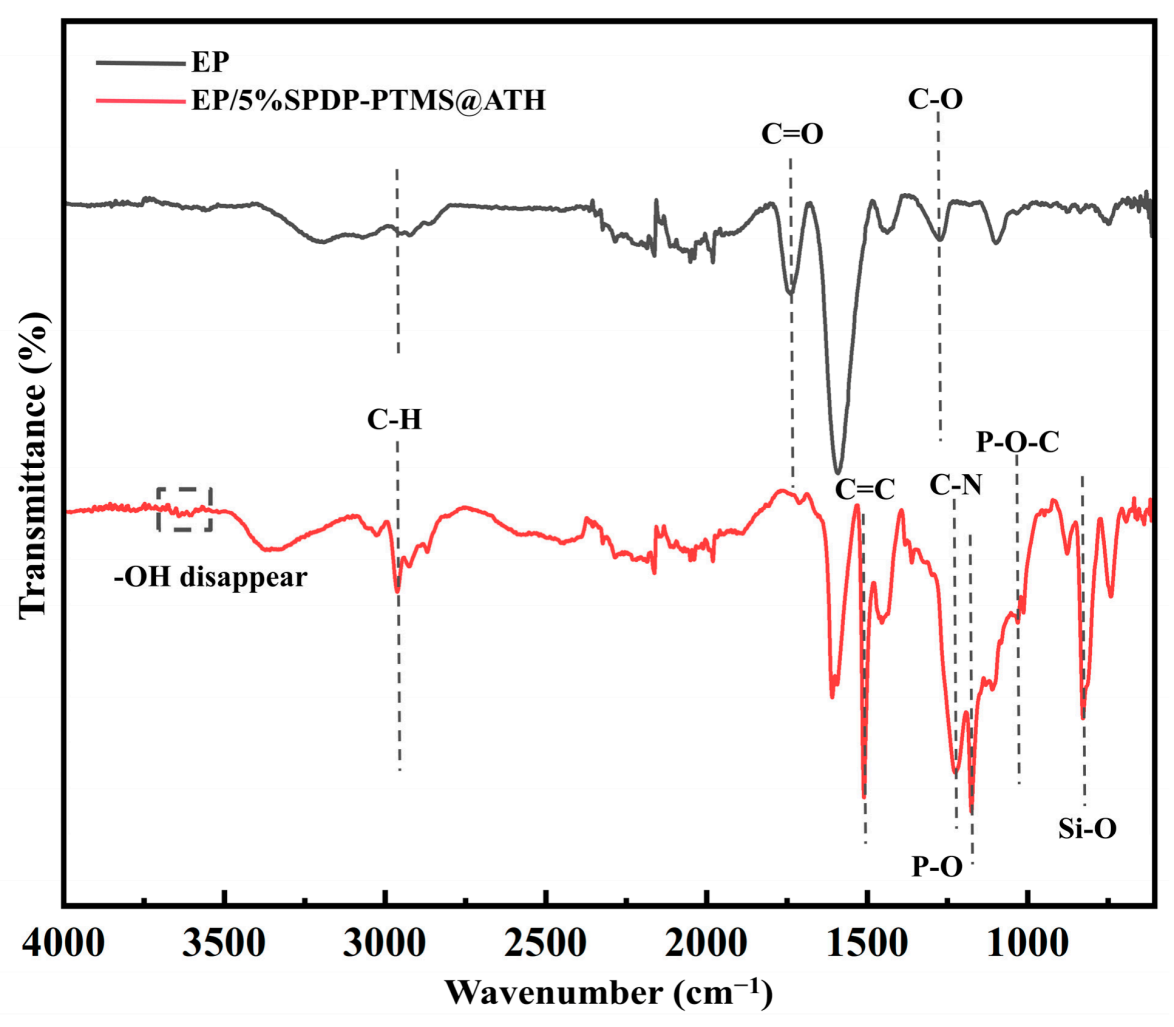
FTIR spectra of carbon residues of EP and 5%EP/SPDP-PTMS@ATH.

FTIR analysis was also performed on the residual char of EP and 5%EP/SPDP-PTMS@ATH after CCT (Figure 9). The intensities of the aromatic C=C (1500 cm^−1^) and C-H (2959 cm^−1^) peaks are strong indicators of char stability [50]. Their stronger peaks imply higher aromatic ring density and fewer aliphatic chains, enhancing resistance to heat and oxygen and thus improving char stability. As shown in Figure 9, the burned EP sample shows almost no peaks of these, whereas 5 wt.% EP/SPDP-PTMS@ATH exhibits pronounced signals, consistent with the improved graphitization observed in the Raman spectra.

In addition, the FTIR spectrum of EP residue reveals a significant C=O absorption at 1739 cm^−1^, indicating the abundant presence of polar oxygen-containing groups (e.g., ester and carboxyl). A strong C-O absorption at 1270 cm^−1^ registers the presence of ether or hydroxyl groups. Since these oxygenated groups are typically eliminated during complete carbonization, their retention indicates that the matrix was only partially carbonized, resulting in reduced char compactness [51]. In contrast, both peaks are significantly weakened or even absent in the 5%EP/SPDP-PTMS@ATH residue, demonstrating that the SPDP-PTMS@ATH suppresses the formation of such oxygenated groups and promotes a denser and more stable char structure.

Furthermore, the Si-O absorption band of SPDP-PTMS@ATH at 1013.88 cm^−1^ (Figure 3a) shifts to 829 cm^−1^ after combustion. This peak shift suggests that the silicon-containing components of the hybrid filler may convert into silicon-aluminum inorganic residues under high-temperature combustion conditions, which enhances crystallinity and reinforces char stability. Meanwhile, the broad -OH absorption band near 3500 cm^−1^ shows obvious attenuation compared with the spectrum of pure SPDP-PTMS@ATH (Figure 3a), which implies that the ATH component may experience endothermic dehydration and release bound water. This dehydration process may lower the surface temperature of the epoxy matrix and generate aluminum oxide species that deposit on the surface of char residues, which potentially provides extra barrier effects against heat and oxygen.

After incorporating SPDP-PTMS@ATH, the char yield increases significantly (Figure 6 and Figure 4c), and FTIR reveals strong C=C (1500 cm^−1^) and P-O (1176 cm^−1^) peaks after combustion. This indicates the successful crosslinking of EP onto the phosphorus-containing aromatics of SPDP-PTMS@ATH. In addition, the simultaneous presence of P-O-C (1031 cm^−1^) and C-N (1230 cm^−1^) peaks indicates the presence of phosphorus-nitrogen heterocyclic structures of SPDP-PTMS@ATH [52,53].

#### 3.3.6. Resultant Gas TGIR Analysis

**Figure 10:**
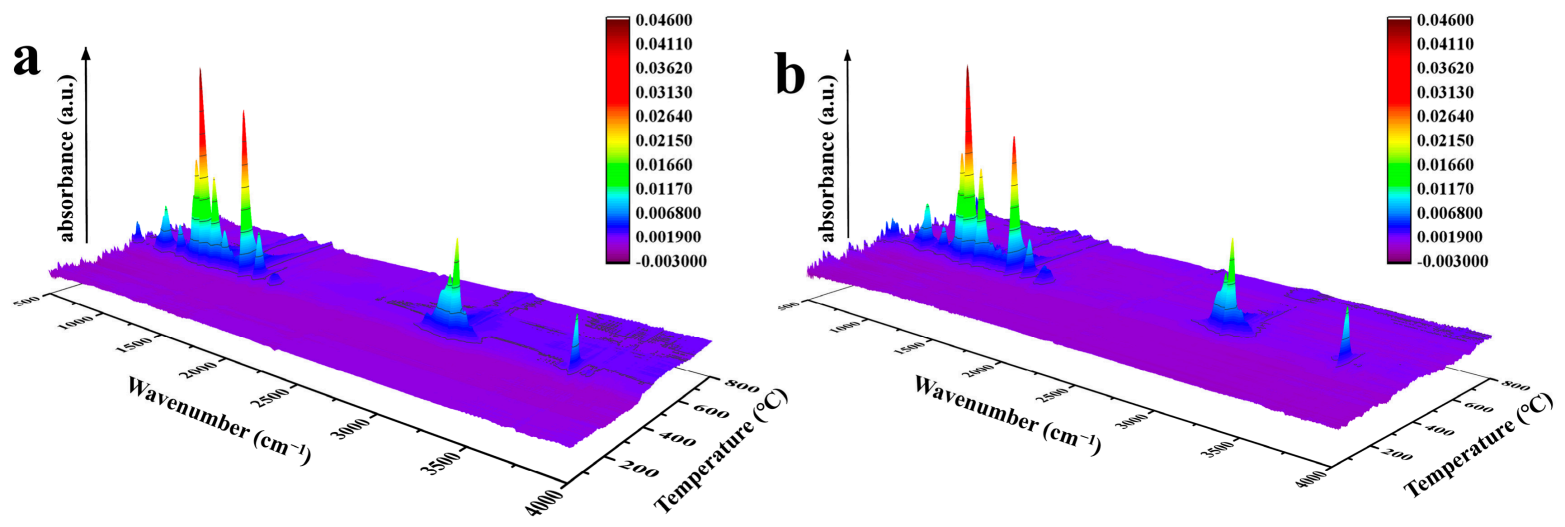
TGIR spectrum of the resultant gas of (**a**) EP and (**b**) EP/5%SPDP-PTMS@ATH during combustion.

**Figure 11:**
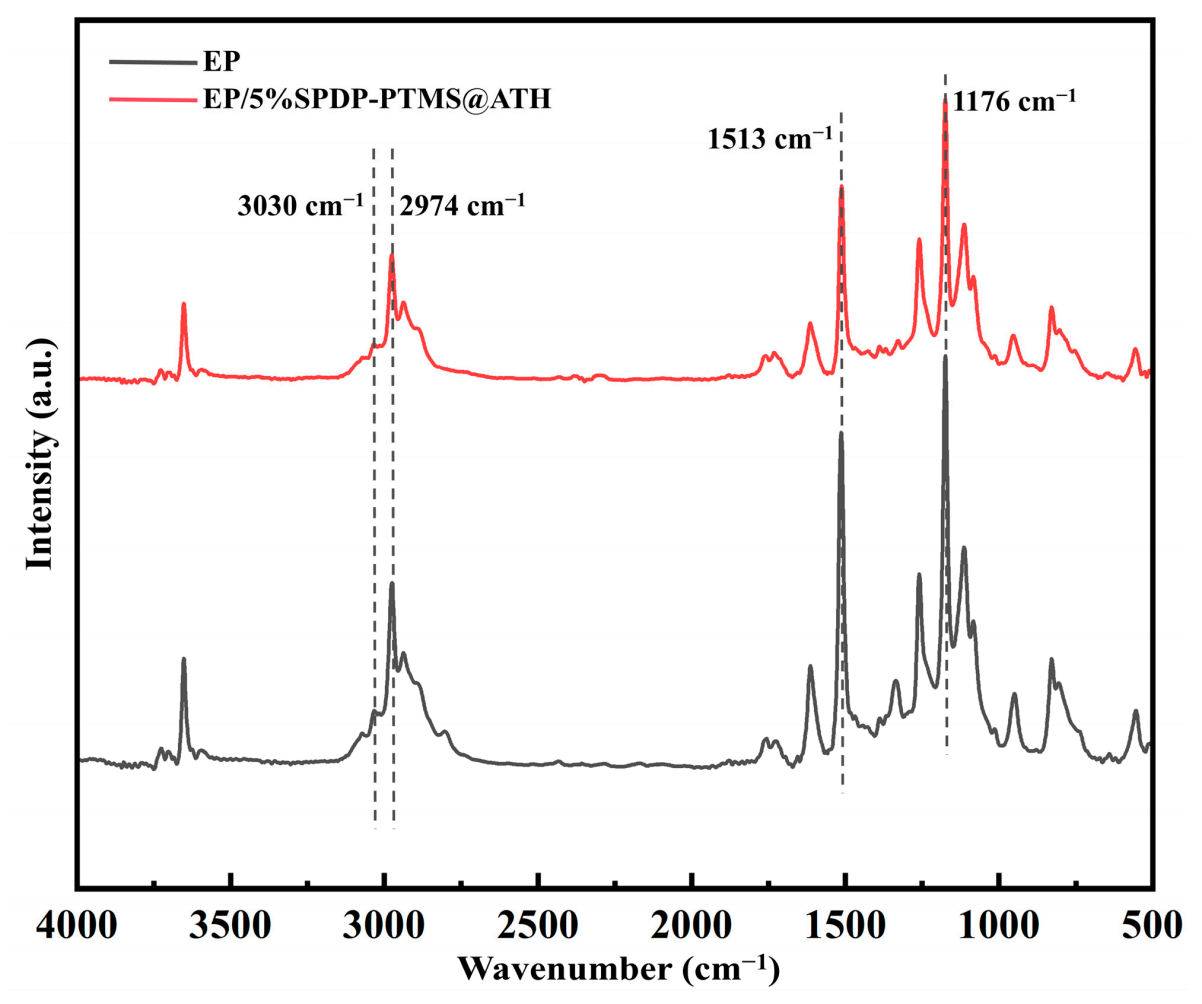
FTIR spectrum of the resultant gas of EP and EP/5%SPDP-PTMS@ATH during combustion.

**Figure 12:**
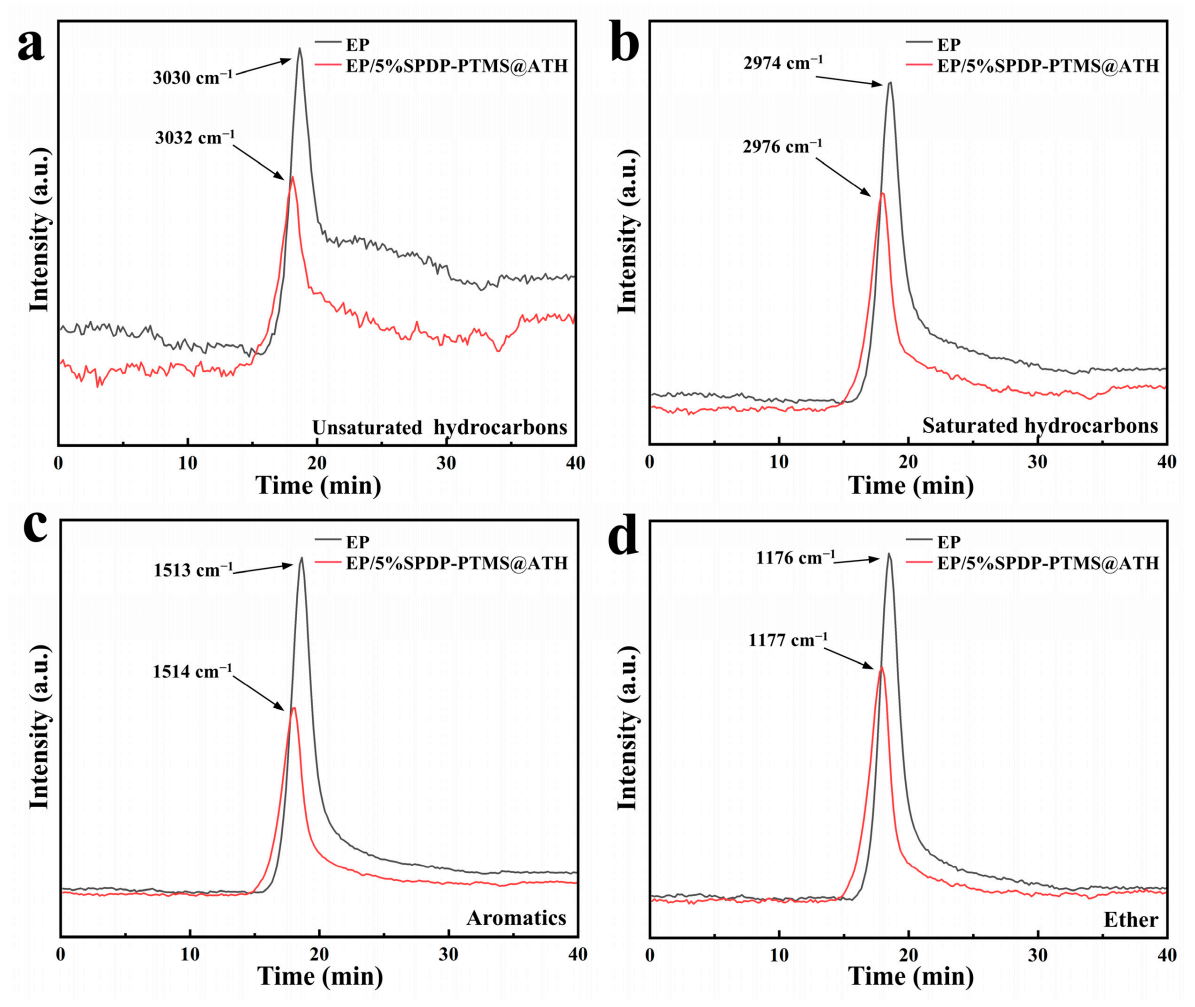
Gaseous product intensities of (**a**) unsaturated hydrocarbons, (**b**) saturated hydrocarbons, (**c**) aromatic compounds, and (**d**) ethers of EP and EP/5%SPDP-PTMS@ATH.

TGIR analysis was performed on the gases released during the combustion of pristine EP and EP/SPDP-PTMS@ATH. The 3D spectra are shown in Figure 10a,b, with the FTIR spectra at maximum degradation rate presented in Figure 11. Figure 12a–d illustrates the release of four typical combustible volatiles: unsaturated hydrocarbons (~3030 cm^−1^), saturated hydrocarbons (~2974 cm^−1^), aromatics (~1513 cm^−1^), and ethers (~1176 cm^−1^) [54].

As seen in Figure 10 and Figure 11, both samples exhibit similar degradation pathways, indicating that the addition of SPDP-PTMS@ATH does not alter the intrinsic decomposition mechanism of EP; this further supports the TG finding that its thermal decomposition trend and rate remain basically unchanged. However, Figure 12 shows that the intensity of combustible gas release is markedly reduced in EP/5%SPDP-PTMS@ATH compared with pristine EP. This confirms that SPDP-PTMS@ATH effectively suppresses volatile fuel release, thereby weakening or interrupting chain reactions in the gas phase. This also corroborates Figure 5d from the cone calorimetry results, confirming that the incorporation of SPDP-PTMS@ATH reduces the total amount of smoke released.

Combined with the cone calorimetry results (Figure 5c,d), where both CO and total smoke release were significantly reduced, the TGIR data further demonstrate that incorporation of SPDP-PTMS@ATH decreases combustible gas emissions and enhances the overall flame-retardant effect.

#### 3.3.7. Flame-Retardant Mechanism Analysis

**Figure 13:**
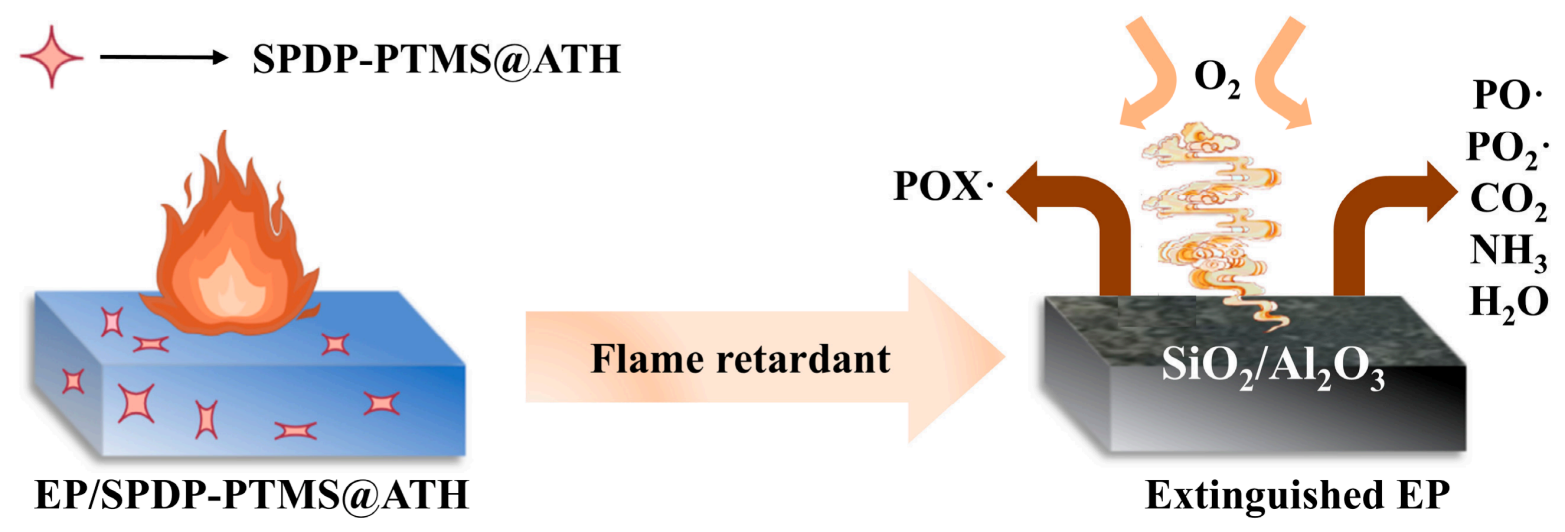
Plausible flame-retardant mechanisms of EP/SPDP-PTMS@ATH.

Based on macroscopic combustion observations and our systematic characterizations (SEM, Raman and FTIR of char residues, as well as TGIR for pyrolysis gases), a distinctive dual-phase synergistic flame-retardant mechanism for EP/SPDP-PTMS@ATH is proposed in Figure 13. In contrast to conventional single P-N or neat ATH additives, this hybrid filler achieves unique multi-element coupling of P, N, Si and Al via coordination between organic phosphorus-silicon chains and inorganic ATH nanoparticles, an interaction seldom reported for epoxy flame retardants.

In the gas phase, thermal cleavage of P-O bonds in grafted SPDP-PTMS yields PO· and PO_2_· radicals to trap H·/OH· and terminate combustion chain reactions. Meanwhile, pyrolysis of nitrogen-containing groups releases inert NH_3_/CO_2_, and ATH decomposes into water vapor; these inert gases dilute oxygen and combustible volatiles to restrain flame propagation. Benefiting from the synergy of P, N, Si, and Al, matrix carbonization is remarkably promoted, lowering toxic CO release and increasing CO_2_ yield to reduce smoke toxicity compared with unmodified ATH or single P-N additives.

In the condensed phase, decomposed P-O groups produce phosphoric/polyphosphoric acids to catalyze epoxy dehydration and char formation. Distinct from traditional mono-component flame retardants, the coupled mineralization of organic P-Si segments and inorganic ATH generates thermally stable SiO_2_ and Al_2_O_3_, which embed inside char to construct a rigid inorganic network. Such hybrid inorganic reinforcement densifies the char layer and prevents crack formation, blocking heat and volatile transfer effectively; this conclusion is well supported by our SEM (compact char morphology) and Raman (high graphitization degree) results. The formed SiO_2_ and Al_2_O_3_ assemble into a low-conductivity inorganic film covering char residues, which blocks vertical heat penetration into the epoxy substrate and accelerates lateral heat dispersion on the char surface to avoid local heat accumulation.

Overall, SPDP-PTMS@ATH exhibits a dual-mode flame-retardant mechanism: gas-phase radical quenching and dilution, combined with condensed-phase char promotion and reinforcement, leading to efficient suppression of combustion in EP composites.

### 3.4. Mechanical Properties

**Figure 14:**
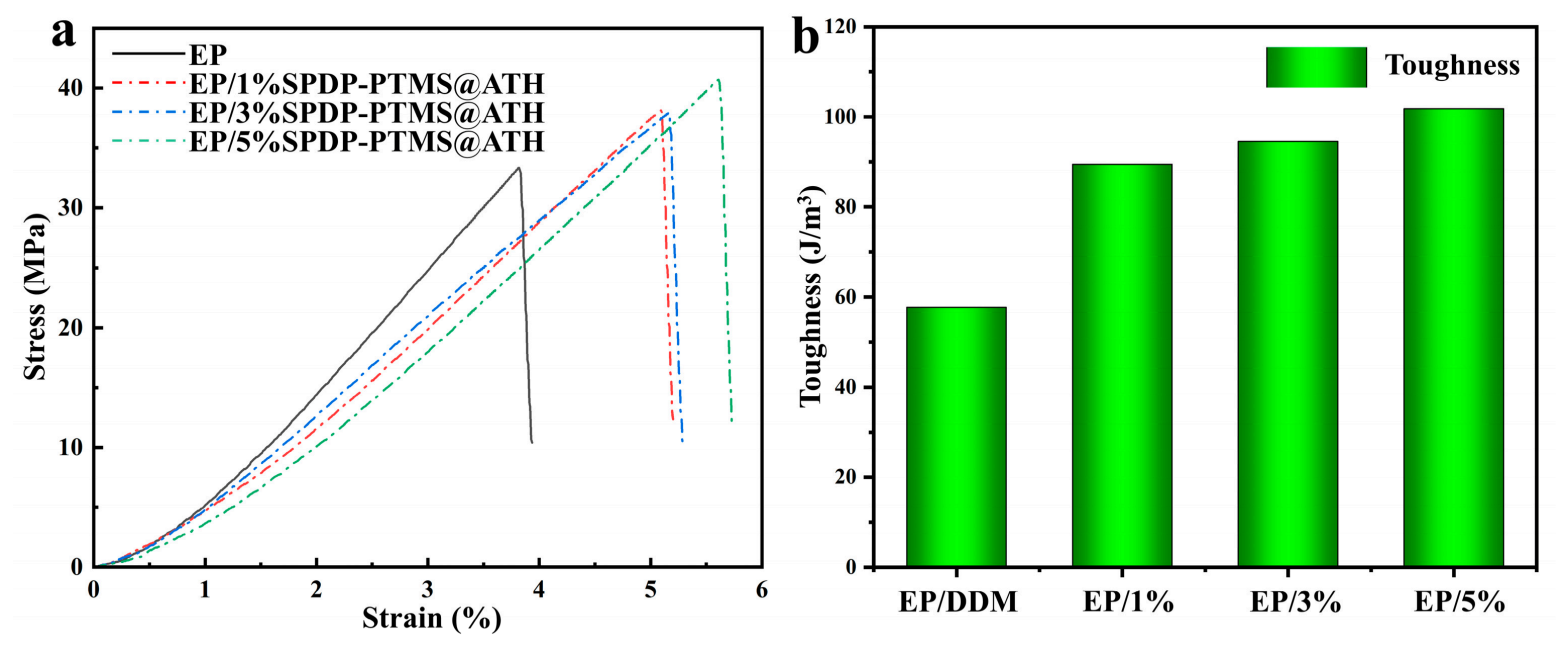
(**a**) Stress and (**b**) strain curves of EP and EP/SPDP-PTMS@ATH.

To evaluate the mechanical properties of the materials, the tensile properties of pristine EP and EP composites with 1, 3, and 5 wt.% SPDP-PTMS@ATH were evaluated (Figure 14 and Table 4). Pristine EP exhibited a tensile strength of 33.36 MPa, elongation at break of 3.81%, and toughness of 57.69 J/m^3^. Incorporation of SPDP-PTMS@ATH improved all three parameters, with strength and elongation increasing progressively with higher loadings. At 5 wt.%, the composite showed the most significant enhancement: tensile strength reached 40.70 MPa (22.0% higher than pristine EP), and elongation at break reached 5.62% (47.5% higher).

These improvements indicate that SPDP-PTMS@ATH effectively enhances the toughness of EP. The effect is attributed to interfacial interactions (van der Waals forces) between the SPDP-PTMS@ATH surface and the epoxy matrix, which strengthen the overall structure. In addition, the long-chain organic segments of SPDP-PTMS contribute to stress dispersion within the matrix, reducing stress concentration and preventing brittle fracture.

### 3.5. Performance Comparison

**Figure 15:**
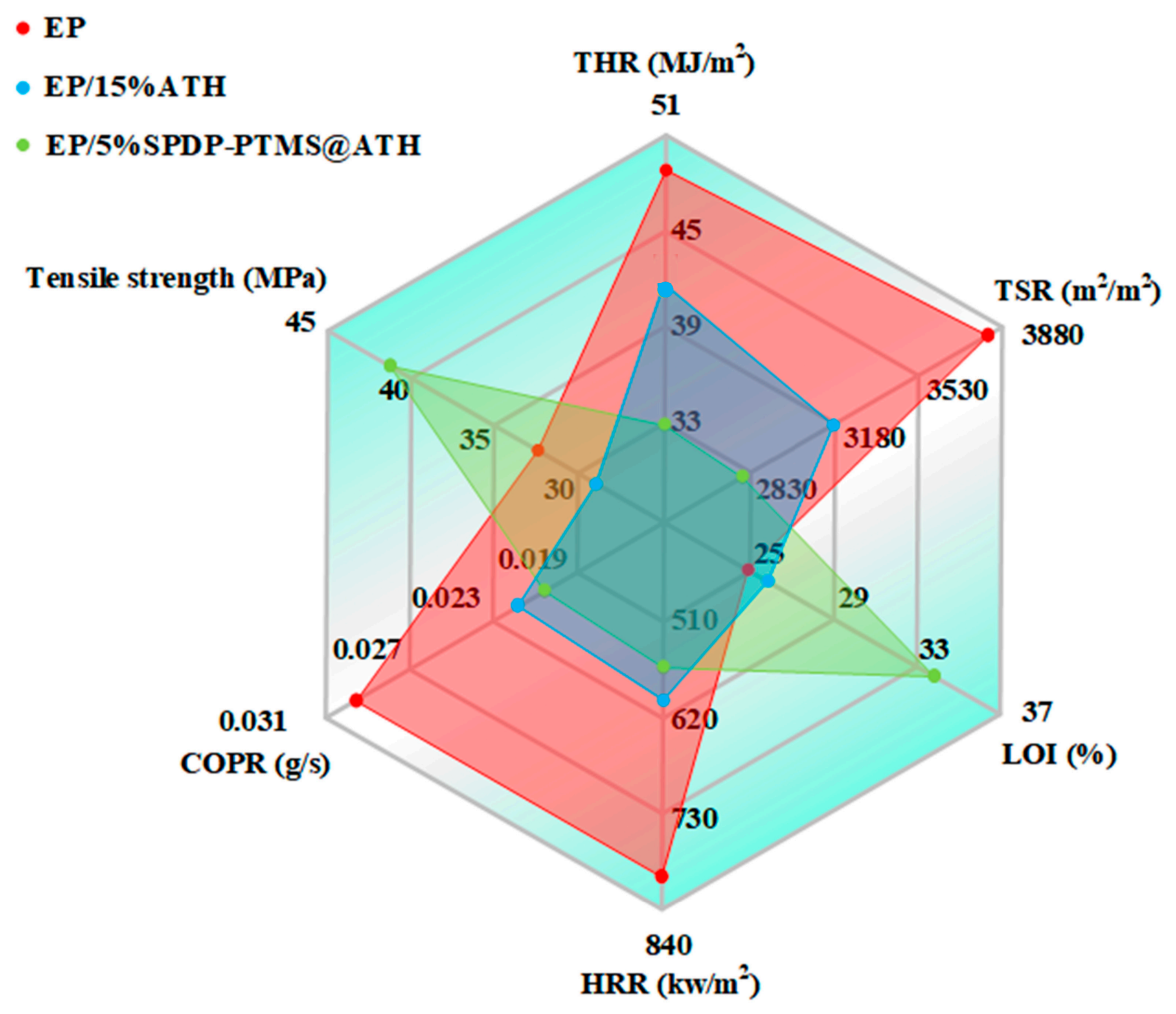
Flame retardancy of EP/5%SPDP-PTMS@ATH compared with EP and EP/15%ATH.

Figure 15 compares the flame-retardant performance of EP composites with unmodified ATH and with SPDP-PTMS@ATH. Incorporation of pure ATH reduced HRR, THR, COPR, and TSR by 28.47%, 14.27%, 30.24%, and 17.65%, respectively, while LOI increased only by 6.51%. However, tensile strength decreased drastically by 51.5%, indicating that although ATH improves flame retardancy, it significantly compromises mechanical properties [55].

In contrast, EP/5%SPDP-PTMS@ATH exhibited HRR, THR, COPR, and TSR reductions of 30.2%, 30.8%, 33.1%, and 26.9%, respectively, while LOI and tensile strength increased by 34% and 22%. These results demonstrate that SPDP-PTMS@ATH not only provides superior flame-retardant efficiency but also enhances the mechanical properties of EP.

Compared with conventional flame retardants, the key advantage of SPDP-PTMS@ATH lies in its ability to achieve balanced property improvements at low loadings. High flame retardancy is obtained without relying on large additive amounts, thereby preserving the inherent characteristics of the matrix. Moreover, the additive simultaneously suppresses HRR, THR, COPR, and TSR, avoiding the trade-offs commonly observed in other systems. This synergy reduces material consumption and formulation complexity, while delivering comprehensive, reliable fire safety across multiple performance dimensions.

## 4. Conclusions

This work successfully synthesized a novel organic–inorganic hybrid flame-retardant SPDP-PTMS@ATH and applied it to modify epoxy resin. In contrast to conventional unmodified ATH and binary phosphorus-aluminum flame retardants reported in most literature, the covalently grafted SPDP-PTMS@ATH exhibits prominent multi-element synergistic effects of N, P, Si, and Al, which effectively improve the fire safety and mechanical performance of epoxy composites simultaneously at low filler loading.

Systematic experimental results demonstrate that SPDP-PTMS@ATH can slightly advance the initial thermal decomposition of epoxy resin, promote early char formation, reduce the thermal degradation rate, and increase the residual char yield at high temperature. The modified EP/5%SPDP-PTMS@ATH composite achieves a significantly improved LOI value of 33.5% and a UL-94 V-0 rating. Cone calorimetry tests confirm that the hybrid filler greatly suppresses heat release, smoke generation, and toxic CO emission, exhibiting superior fire and smoke inhibition performance. Macroscopic and microscopic char analyses verify that SPDP-PTMS@ATH facilitates the formation of dense, continuous, and highly graphitized intumescent char layers, constructing a stable condensed-phase barrier to block heat and mass transfer. Meanwhile, TGIR results confirm that the hybrid flame retardant effectively reduces the release of flammable hydrocarbon, aromatic, and ether volatiles, thereby inhibiting the free-radical chain combustion reaction in the gas phase. Notably, different from traditional flame retardants that deteriorate mechanical properties, SPDP-PTMS@ATH simultaneously enhances the tensile strength and elongation at break of epoxy resin by 22.0% and 47.5%, respectively.

Within the tested gradient of 1–5 wt.%, 5 wt.% is verified as the optimal filler content for this epoxy system. The flame retardancy, char quality, and mechanical properties continuously improve with increasing SPDP-PTMS@ATH loading and reach the best comprehensive performance at 5 wt.%. Further increasing the filler dosage cannot provide additional performance promotion, indicating that the composite system achieves a saturated and balanced performance state at 5 wt.%.

Overall, the designed SPDP-PTMS@ATH breaks the common trade-off between flame retardancy and mechanical properties of traditional ATH-based epoxy materials. Benefiting from the synergistic gas-condensed dual-phase flame-retardant mechanism, the as-prepared epoxy composites achieve high-efficiency fire suppression, low smoke toxicity, and enhanced mechanical robustness at low loading. This work provides a new strategy for the design and preparation of high-performance, balanced, low-loading intumescent flame-retardant epoxy materials, and shows promising application potential in fire-safe coatings, building decorative materials, and lightweight structural composites requiring both mechanical reliability and fire resistance.

## Figures and Tables

**Table 1:** TG analysis data of SPDP-PTMS@ATH, EP and EP composites under N_2_.

Sample	T_5%_ (°C)	T_max_ (°C)	R_max_ (Wt/°C)	CY_800_ (Wt.%)	CY_theoretical_ (Wt%)
SPDP-PTMS@ATH	285.6	295.9	0.22	74.8	-
EP	373.0	399.9	1.37	15.8	-
EP/1%SPDP-PTMS@ATH	372.7	396.1	1.50	22.0	16.39
EP/3%SPDP-PTMS@ATH	371.8	396.1	1.35	29.1	17.57
EP/5%SPDP-PTMS@ATH	341.2	385.2	1.19	32.0	18.75

**Table 2:** LOI and UL-94 test results of EP/SPDP-PTMS@ATH.

Sample	LOI	UL-94	Dripping
EP	25	NR	Yes
EP/1%SPDP-PTMS@ATH	27.3	V-1	No
EP/3%SPDP-PTMS@ATH	29.8	V-1	No
EP/5%SPDP-PTMS@ATH	33.5	V-0	No

**Table 3:** Cone calorimeter data for EP and EP/SPDP-PTMS@ATH.

Sample	TTI (s)	HRR (kW/m^2^)	THR (MJ/m^2^)	COPR (g/s)	TSR (m^2^/m^2^)	CO_2_PR (g/s)	SPR (m^2^/s)
EP	76	814	49.4	0.0296	3870	0.57	0.40
EP/1%SPDP-PTMS@ATH	75	710	44.8	0.0223	3276	0.49	0.38
EP/3%SPDP-PTMS@ATH	80	680	42.9	0.0231	2979	0.45	0.36
EP/5%SPDP-PTMS@ATH	75	568	34.2	0.0198	2830	0.42	0.34

**Table 4:** Tensile properties of EP and EP/SPDP-PTMS@ATH.

Sample	Tensile Modulus (GPa)	Tensile Strength (MPa)	Elongation at Break (%)	Toughness (J/m^3^)
EP	0.876	33.36	3.81	57.69
EP/1%SPDP-PTMS@ATH	0.739	38.14	5.16	89.47
EP/3%SPDP-PTMS@ATH	0.711	37.97	5.34	94.57
EP/5%SPDP-PTMS@ATH	0.724	40.70	5.62	101.83

## Data Availability

The original contributions presented in this study are included in the article. Further inquiries can be directed to the corresponding authors.

## References

[1] Sut A., Greiser S., Jäger C., Schartel B. (2017). Synergy in flame-retarded epoxy resin. J. Therm. Anal. Calorim..

[2] Zhang H., Gong X., Li Z., Wang Y. (2021). Synthesis of new phosphorous-containing flame retardant and the properties of flame retardant epoxy resins. Pigment. Resin Technol..

[3] Zhang J., Wu W., Lv Y., Guo C., Xu Y., Zeng B., Dai L. (2021). Grafting multi-elements hybrid polymer brushes on graphene oxide for epoxy composite with excellent flame retardancy. J. Appl. Polym. Sci..

[4] Zhang Y.-C., Xu G.-L., Liang Y., Yang J., Hu J. (2016). Preparation of flame retarded epoxy resins containing DOPO group. Thermochim. Acta.

[5] Goyat M., Hooda A., Gupta T.K., Kumar K., Halder S., Ghosh P., Dehiya B.S. (2021). Role of non-functionalized oxide nanoparticles on mechanical properties and toughening mechanisms of epoxy nanocomposites. Ceram. Int..

[6] Li X., Xie Q., Lin J., Huang J., Xie K., Liu J., Li X., Wei W. (2023). A phosphorus/silicon-containing flame retardant based on eugenol for improving flame retardancy and smoke suppression of epoxy resin. J. Appl. Polym. Sci..

[7] Meng W., Wu H., Bi X., Huo Z., Wu J., Jiao Y., Xu J., Wang M., Qu H. (2021). Synthesis of ZIF-8 with encapsulated hexachlorocyclotriphosphazene and its quenching mechanism for flame-retardant epoxy resin. Microporous Mesoporous Mater..

[8] Fu X.-L., Ding H.-L., Wang X., Song L., Hu Y. (2022). Fabrication of zirconium phenylphosphonate/epoxy composites with simultaneously enhanced mechanical strength, anti-flammability and smoke suppression. Compos. Part A Appl. Sci. Manuf..

[9] Xu L., Lei C., Xu R., Zhang X., Zhang F. (2016). Functionalization of α-zirconium phosphate by polyphosphazene and its effect on the flame retardance of an intumescent flame retardant polypropylene system. RSC Adv..

[10] Wu Y., Yang T., Cheng Y., Huang T., Yu B., Wu Q., Zhu M., Yu H. (2023). Synthesis phosphorus–sulfur reactive flame retardant for polyamide 66 with high flame retardant efficiency and low smoke. Polym. Degrad. Stab..

[11] Chen H., Zhu S., Zhou R., Wu X., Zhang W., Han X., Wang J. (2022). Thermal Degradation Behavior of Thiol-ene Composites Loaded with a Novel Silicone Flame Retardant. Polymers.

[12] Zhang T., Liu W., Wang M., Liu P., Pan Y., Liu D. (2016). Synergistic effect of an aromatic boronic acid derivative and magnesium hydroxide on the flame retardancy of epoxy resin. Polym. Degrad. Stab..

[13] Dowbysz A., Samsonowicz M., Kukfisz B. (2021). Modification of glass/polyester laminates with flame retardants. Materials.

[14] Zhu D., Bi Q., Yin G.-Z., Jiang Y., Fu W., Wang N., Wang D.-Y. (2022). Investigation of magnesium hydroxide functionalized by polydopamine/transition metal ions on flame retardancy of epoxy resin. J. Therm. Anal. Calorim..

[15] He X., Guan J., Chen Z., Cao Z., Li Y., Lei Z., Chen D. (2023). Preparation of a novel intrinsic flame retardant epoxy resin based on L-arginine functionalized magnesium hydroxide. Eur. Polym. J..

[16] Pu Z., Zou L., Wu F., Wang X., Peng Q., Zhong J., Liu X., Wang Y., Pan Y., Jiang D. (2023). Preparation of phosphorus-nitrogen flame retardant DOPO-DBAPh and its flame retarded epoxy-based composites. J. Vinyl Addit. Technol..

[17] Yuan Z., Wen H., Liu Y., Wang Q. (2021). Synergistic effect between piperazine pyrophosphate and melamine polyphosphate in flame retarded glass fiber reinforced polypropylene. Polym. Degrad. Stab..

[18] Zhu S., Gong W., Luo J., Meng X., Xin Z., Wu J., Jiang Z. (2019). Flame Retardancy and Mechanism of Novel Phosphorus-Silicon Flame Retardant Based on Polysilsesquioxane. Polymers.

[19] Yu M., Zhang T., Li J., Tan J., Zhu X. (2023). Synthesis of a Multifunctional Phosphorus/Silicon Flame Retardant via an Industrial Feasible Technology. ACS Sustain. Chem. Eng..

[20] Xie Y., Liu C., Wang Y., Bao D., Yan W., Zhou G. (2024). Waterborne Polyurethane Treated with Flame Retardant Based on Polydimethylsiloxanes and Boron Phenolic Resin for Improving the Char Residue and Anti-Dripping Performance. Molecules.

[21] Liu Z., Ma M., Ge B., Zheng Y., Chen S., Shi Y., He H., Zhu Y., Wang X. (2023). Toward flame-retardant, transparency, and high mechanical property of polycarbonate based on low addition of linear polyborosiloxane. Chem. Eng. J..

[22] Li G., Duan B., Leng G., Xiu X., Wang S., Li S., Zhao W., Liu J., Qu J. (2025). Preparation of Nitrogen-Phosphorus-Silicon Synergistic Intumescent Flame Retardant and Its Effect on the Flame Retardancy of Waterborne Polyurethane. J. Appl. Polym. Sci..

[23] Liu C., Huang K., Liu R., Li Y., Dai L., Wang W. (2022). A multi-element flame retardant on the basis of silicon-phosphorus-nitrogen for combustibility suppressing of epoxy. Polym. Test..

[24] Wang S., Huang J., Chen F. (2012). Study on Mg-Al hydrotalcites in flame-retardant paper preparation. BioResources.

[25] Yan L., Xu Z., Wang X., Deng N., Chu Z. (2018). Synergistic effects of aluminum hydroxide on improving the flame retardancy and smoke suppression properties of transparent intumescent fire-retardant coatings. J. Coat. Technol. Res..

[26] Zhang S., Li Y., Guo J., Gu L., Li H., Fei B., Sun J., Gu X. (2019). Preparation of hexakis (4-aldehyde phenoxy) cyclotriphosphazene grafted kaolinite and its synergistic fire resistance in poly (butylene succinate). Polym. Compos..

[27] Li Y., Li N., Shen M., Cao L., Ling J., Xu Q., Qu J., Fan Y. (2023). Synthesis of organic–inorganic hybrid flame retardant and its use in polyurethane composite fiber membrane. J. Appl. Polym. Sci..

[28] Yu H., Xia Y., Xu X., Zarshad N., Wu M., Ni H. (2020). Preparation of organic–inorganic intumescent flame retardant with phosphorus, nitrogen and silicon and its flame retardant effect for epoxy resin. J. Appl. Polym. Sci..

[29] Liu J., Dong C., Zhang Z., Sun H., Kong D., Lu Z. (2020). Durable flame retardant cotton fabrics modified with a novel silicon–phosphorus–nitrogen synergistic flame retardant. Cellulose.

[30] Tieves F., Tonin F., Fernández-Fueyo E., Robbins J.M., Bommarius B., Bommarius A.S., Alcalde M., Hollmann F. (2019). Energising the E-factor: The E^+^-factor. Tetrahedron.

[31] Song K., Wang Y., Ruan F., Yang W., Liu J. (2020). Synthesis of a Novel Spirocyclic Inflatable Flame Retardant and Its Application in Epoxy Composites. Polymers.

[32] Song K., Wang Y., Ruan F., Liu J., Li N., Li X. (2020). Effects of a Macromolecule Spirocyclic Inflatable Flame Retardant on the Thermal and Flame Retardant Properties of Epoxy Resin. Polymers.

[33] Zhang X., Wang Z., Xie J. (2023). Synthesis and curing properties of fluorinated curing agent for epoxy resin based on natural soybean isoflavones. React. Funct. Polym..

[34] Yu S., Li X., Zou M., Guo X., Ma H., Wang S. (2020). Effect of the Aromatic Amine Curing Agent Structure on Properties of Epoxy Resin-Based Syntactic Foams. ACS Omega.

[35] Shi J., Zeng W., Yang Z., Li J., Zhao P., Li H., Yan X., Wen N., Lei Z., Chen D. (2021). Effect of particle size on flame retardancy and mechanical properties of hydroxyethyl diphosphate modified aluminum hydroxide intrinsic polyethylene terephthalate. J. Appl. Polym. Sci..

[36] Vejayan H., Gutiérrez-González A., Torio M.E., Busnengo H.F., Beck R.D. (2022). Methylidyne Adsorption on Pt(211) Probed by Reflection Absorption Infrared Spectroscopy (RAIRS). J. Phys. Chem. C.

[37] Zhang H.T., Sun Z.X., Hu Y.H. (2021). Steam Reforming of Methane: Current States of Catalyst Design and Process Upgrading. Renew. Sustain. Energ. Rev..

[38] Torio M.E., Busnengo H.F. (2020). Site-Specific Product Selectivity of Stepped Pt Surfaces for Methane Dehydrogenation. J. Phys. Chem. C.

[39] Mitsui T., Matsui T., Kikuchi R., Eguchi K. (2009). Microstructural Transformation with Heat-Treatment of Aluminum Hydroxide with Gibbsite Structure. Bull. Chem. Soc. Jpn..

[40] Ning X. (2018). Kinetics of pyrolysis of high purity ultrafine aluminum hydroxide. Hunan Non-Ferr. Met..

[41] Yang W.L., Li M., Zhang Y., Liu X.Y. (2025). A Novel Organic-Inorganic PSi-NH2 Hybrid Flame Retardant and Its Effects on Flame Retardancy and Mechanical Properties of Polylactic Acid. J. Appl. Polym. Sci..

[42] Yang J., Hua Y., Sun J., Wang X., Gu X., Zhang S. (2023). A Boron-Containing Ammonium Polyphosphate for Epoxy Resins with Enhanced Flame Retardancy and Reduced Smoke Release. Adv. Eng. Mater..

[43] Zhang W., Fina A., Ferraro G., Yang R. (2018). FTIR and GCMS analysis of epoxy resin decomposition products feeding the flame during UL 94 standard flammability test. Application to the understanding of the blowing-out effect in epoxy/polyhedral silsesquioxane formulations. J. Anal. Appl. Pyrolysis.

[44] Li S., Yi D.Q., Lu H.Y., Hao J.W. (2025). All-In-One 2D Intumescent Flame Retardants [Fe(OH)_2_]^+^ Anchored Melamine Trimetaphosphate (Fe@MAP) Enable Enhanced Flame Retardancy and Smoke Suppression of Epoxy Resins. J. Appl. Polym. Sci..

[45] Vahabi H., Movahedifar E., Kandola B.K., Saeb M.R. (2023). *Flame Retardancy Index* (*FRI*) for Polymer Materials Ranking. Polymers.

[46] Wang J., Ma C., Mu X., Zhou X., He L., Xiao Y., Song L., Hu Y. (2020). Designing 3D ternary-structure based on SnO_2_ nanoparticles anchored hollow polypyrrole microspheres interconnected with N, S co-doped graphene towards high-performance polymer composite. Chem. Eng. J..

[47] Zhang Z., Li X., Ma Z., Ning H., Zhang D., Wang Y. (2020). A facile and green strategy to simultaneously enhance the flame retardant and mechanical properties of poly(vinyl alcohol) by introduction of a bio-based polyelectrolyte complex formed by chitosan and phytic acid. Dalton Trans..

[48] Huo S., Yang S., Wang J., Cheng J., Zhang Q., Hu Y., Ding G., Zhang Q., Song P. (2020). A liquid phosphorus-containing imidazole derivative as flame-retardant curing agent for epoxy resin with enhanced thermal latency, mechanical, and flame-retardant performances. J. Hazard. Mater..

[49] Zhang L., Wang Q., Jian R.-K., Wang D.-Y. (2020). Bioinspired iron-loaded polydopamine nanospheres as green flame retardants for epoxy resin *via* free radical scavenging and catalytic charring. J. Mater. Chem. A.

[50] Jiang H., Jie L., Zhang L., Li S.X., Tang T., Wang S. (2024). Preparation and properties of low smoke, low heat and thin wall flame retardant polycarbonate materials. Acta Mater. Compos. Sin..

[51] Xu Z.Y., Hou Z.M., Ye X.L., Qi Y.Z., Xu S.J., Bao D.M., Zhang D.H., Zhou G.Y., Cai X.D., Zou G.L. (2023). Study on the performance of HPCTP-DOPS/EP, a double-base synergistic flame retardant epoxy resin. Acta Mater. Compos. Sin..

[52] Zhu Z.-M., Wang L.-X., Lin X.-B., Dong L.-P. (2019). Synthesis of a novel phosphorus-nitrogen flame retardant and its application in epoxy resin. Polym. Degrad. Stab..

[53] Lu W., Jin Z. (2022). Synthesis of phosphorus/nitrogen containing intumescent flame retardants from p-hydroxybenzaldehyde, vanillin and syringaldehyde for rigid polyurethane foams. Polym. Degrad. Stab..

[54] Gou Y., Zha Y.-F., Sun D.-X., Yang J.-H., Wang Y. (2025). Synchronously enhanced flame-retardance and mechanical properties of epoxy resin via a P/N-containing active curing agent. Polymer.

[55] Chai G., Zhu G., Gao S., Zhou J., Gao Y., Wang Y. (2019). On improving flame retardant and smoke suppression efficiency of epoxy resin doped with aluminum tri-hydroxide. Adv. Compos. Lett..

